# When less is more – a fast TurboID knock-in approach for high-sensitivity endogenous interactome mapping

**DOI:** 10.1242/jcs.261952

**Published:** 2024-08-28

**Authors:** Alexander Stockhammer, Carissa Spalt, Antonia Klemt, Laila S. Benz, Shelly Harel, Vini Natalia, Lukas Wiench, Christian Freund, Benno Kuropka, Francesca Bottanelli

**Affiliations:** ^1^ Membrane Trafficking Laboratory, Institute for Chemistry and Biochemistry, Freie Universität Berlin, Thielallee 63, 14195 Berlin, Germany; ^2^ Laboratory of Protein Biochemistry, Institute for Chemistry and Biochemistry, Freie Universität Berlin, Thielallee 63, 14195 Berlin, Germany

**Keywords:** Gene editing, TurboID, Membrane trafficking

## Abstract

In recent years, proximity labeling has established itself as an unbiased and powerful approach to map the interactome of specific proteins. Although physiological expression of labeling enzymes is beneficial for the mapping of interactors, generation of the desired cell lines remains time-consuming and challenging. Using our established pipeline for rapid generation of C- and N-terminal CRISPR-Cas9 knock-ins (KIs) based on antibiotic selection, we were able to compare the performance of commonly used labeling enzymes when endogenously expressed. Endogenous tagging of the µ subunit of the adaptor protein (AP)-1 complex with TurboID allowed identification of known interactors and cargo proteins that simple overexpression of a labeling enzyme fusion protein could not reveal. We used the KI strategy to compare the interactome of the different AP complexes and clathrin and were able to assemble lists of potential interactors and cargo proteins that are specific for each sorting pathway. Our approach greatly simplifies the execution of proximity labeling experiments for proteins in their native cellular environment and allows going from CRISPR transfection to mass spectrometry analysis and interactome data in just over a month.

## Teaser

**Summary:** A fast CRISPR-pipeline to endogenously tag proteins of interest with labeling enzymes on their C- and N-terminus; this was used to map the interactome of different adaptor protein complexes using TurboID.

## INTRODUCTION

The biological role of a protein is shaped by its subcellular localization and its interaction with other biomolecules. Therefore, mapping the interactors of a given protein can be crucial for understanding its biological function. In the past several years, proximity labeling with biotin in living cells has emerged as a complementary approach to classic affinity purification-mass spectrometry (MS)-based methods for mapping of protein–protein interactions in living cells and organisms (Qin et al., 2021; Trinkle-Mulcahy, 2019). The proximity labeling is carried out by enzymes genetically fused to the protein of interest (POI) that catalyze the formation of a highly reactive biotin intermediate labeling proteins within a small radius (1–10 nm) (Kim et al., 2014; Martell et al., 2012) in a promiscuous manner. A key advantage of proximity labeling-based interactome mapping compared to traditional approaches is that very weak and transient interactions can also be captured. The biotinylation itself provides a unique chemical moiety that can be used for subsequent enrichment and identification.

The enzymes used for proximity labeling are either engineered peroxidases [APEX (Rhee et al., 2013), APEX2 (Lam et al., 2015)] or engineered biotin ligases [BioID (Roux et al., 2012), BioID2 (Kim et al., 2016), TurboID (Branon et al., 2018) and miniTurboID (Branon et al., 2018)]. APEX and APEX2 use H_2_O_2_ as a co-substrate to rapidly generate a highly reactive phenoxyl radical from biotin-phenol that reacts specifically with electron-rich side chains (primarily tyrosine) (Branon et al., 2018). An attractive feature of APEX peroxidases is the fast labeling kinetics (labeling time: <1 min) that enable probing with a high temporal resolution. However, on the downside, they require H_2_O_2_, which causes oxidative stress in living cells and thus cannot be used for proximity labeling in living organisms. In contrast, biotin ligases simply need non-toxic, highly soluble biotin as a substrate which, in an ATP-dependent reaction, is converted into a reactive biotinoyl-5′-AMP intermediate that covalently tags proximal lysine residues (Roux et al., 2012). Although the labeling time can be reduced from >18 h for BioID (Roux et al., 2012) to less than an hour with TurboID (Branon et al., 2018), labeling with biotin ligases is significantly slower than with APEX peroxidases.

To avoid artifacts as a result of overexpression, such as mislocalization and protein aggregation among others (Gibson et al., 2013; Moriya, 2015; Rizzo et al., 2009), physiological expression levels of the fusion protein are preferred for interactome mapping experiments. Moreover, less abundant interactors that would be hidden in the unspecific labeling background, resulting from non-physiological expression levels, might not be identified this way. To control expression levels of the fusion protein overall, various different approaches have been implemented. These include the use of small-molecule-induced expression systems in a safe DNA locus (such as the Flp-In™ T-Rex™ system) (Kim et al., 2021; Lambert et al., 2015), CRISPR-Cas9-based genome editing (Gupta et al., 2018; Luciano et al., 2022), or knockout and replacement approaches, in which the gene is first knocked out and then the fusion protein is integrated in a safe locus (Chua et al., 2021). However, these methods either require the time-consuming selection and testing of single cell clones (CRISPR-based approaches) or are limited to standardized commercially available cell lines and non-endogenous protein expression (inducible expression systems).

Here, we combine a rapid knock-in (KI) strategy for C- or N-terminal tagging based on antibiotic selection of positively edited cells (Bottanelli et al., 2017; Schmidt et al., 2016) with proximity-based proteomics to detect interactors at physiological expression levels. This versatile approach enabled us to endogenously tag proteins with the four most commonly used labeling enzymes (APEX2, BioID2, miniTurboID and TurboID) and compare their performance when expressed endogenously. In the past, proximity biotinylation approaches have been instrumental to map the surface proteome of membrane-bound organelles and the interactome of membrane-associated proteins (Antonicka et al., 2020; Banerjee et al., 2022; Go et al., 2021; Hesketh et al., 2020; Liu et al., 2018). Therefore, we tagged the µ1A subunit of the adaptor protein complex AP-1 (AP1µA, encoded by *AP1M1*) at the C-terminus and clathrin light chain (CLC, specifically CLCa, encoded by *CLTA*) at the N-terminus. AP-1, together with CLC, mediates transport of specific cargo between the trans-Golgi network (TGN) and endosomes (Hirst and Robinson, 1998; Ren et al., 2013) and transiently associates to membranes, leaving excess, non-membrane bound AP-1 subunits and CLC in the cytosol. Our data reveals that endogenous tagging allows proximity labeling with higher specificity compared to simple overexpression of the labeling enzyme. Known interactors of AP-1, as well as specific cargo, were more highly enriched or even exclusively found in the experiments performed with a KI cell line. We identified TurboID to be best suited for KI proximity labeling, and propose a pipeline for rapid endogenous tagging to improve the workflow and quality of proximity-based MS experiments. The use of this pipeline allowed us to compare the interactome of different AP complexes, including the low-abundance AP-4, and assemble a list of potential interactors and cargo proteins for each adaptor protein complex. Finally, we compared the interactome of CLCa to AP-1 and AP-2, which are both clathrin adaptors, to show that the here presented KI-strategy with TurboID allows identification of pathway specific interactors.

## RESULTS

### Endogenous C-terminal tagging of AP1µA with labeling enzymes

**Fig. 1.**
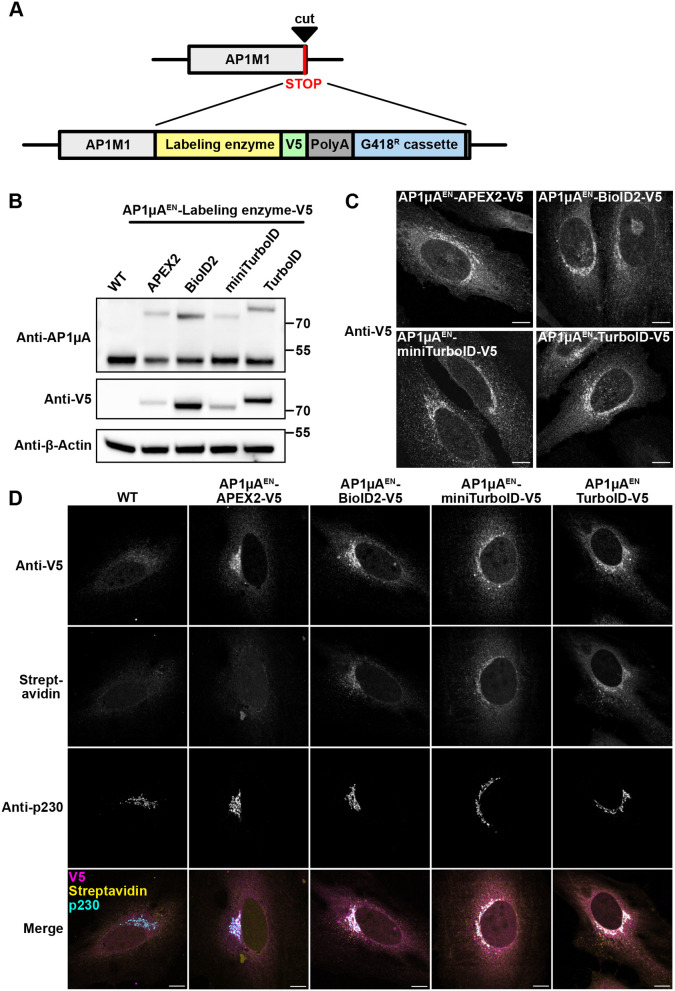
**Rapid KI-strategy allows for endogenous tagging of AP1µA with different labeling enzyme for proximity biotinylation.** (A) Scheme of the KI strategy. AP1µA was C-terminally tagged with the labeling enzyme, a V5 tag and a resistance cassette (including promoter, resistance and termination sequence) that allows for rapid selection of positive cells. (B) Blots of whole-cell lysates from generated cell lines to verify the KI of the labeling enzymes by anti-AP1µA and anti-V5 blotting. (C) Cell lines expressing the labeling enzymes endogenously were fixed and stained with an anti-V5 antibody to detect the labeling enzyme expression and localization. (D) Cells endogenously expressing the different labeling enzymes were fixed and stained with anti-V5 antibody to detect the labeling enzymes, an anti-p230 antibody to mark the TGN area and streptavidin–AF488 to detect biotinylated proteins. Cells were treated with 50 μM biotin for 24 h and AP1μA^EN–^APEX2-V5-expressing cells were incubated for 30 min with 500 μM biotin-phenol and labeling was induced for 1 min with H_2_O_2_. EN, endogenous. All images representative of three repeats. Scale bars: 10 µm.

To evaluate the performance of commonly used labeling enzymes, we used the CRISPR-Cas9 system to genetically fuse APEX2, BioID2, miniTurboID and TurboID to the C-terminus of our chosen target AP1µA in HeLa cells. The insertion of a geneticin (G418) resistance cassette downstream of a polyadenylation signal into the targeted gene locus (*AP1M1*) allowed us to rapidly select for cells that were successfully modified (Bottanelli et al., 2017). In addition, a V5 tag was inserted for KI validation via western blotting and immunofluorescence. The CRISPR strategy is illustrated in Fig. 1A. Cells were transfected with plasmids encoding for a gRNA targeting the genomic locus and homology repair plasmids containing sequences of the various labeling enzymes. At 3 days post transfection, G418 was added, and cells were allowed to grow back to confluency to perform downstream analysis. Expression of the fusion proteins in the four KIs was validated with western blotting and immunofluorescence (Fig. 1B,C). Homology-directed repair is a rare event, mainly yielding a mixed population of heterozygous cells; therefore, we assume most of the generated cells are heterozygous. To estimate the percentage of KI cells that express the fusion protein, we checked for V5 expression using immunofluorescence microscopy to detect positively edited cells and found that 43% (APEX2), 63% (BioID2), 45% (MiniTurboID), 59% (TurboID) of the cells expressed the fusion protein.

Next, we wanted to compare the performance of the various labeling enzymes when expressed under the endogenous promoter. For biotin ligases, labeling times between less than 1 h (miniTurboID and TurboID) (Branon et al., 2018; Cho et al., 2020) and at least 16 h–24 h (BioID2) (Kim et al., 2016; Zhang et al., 2019) are reported. To ensure the amount of biotinylation is sufficient for visualization and comparable between the different biotin ligases, we treated all cell lines with 50 µM biotin for 24 h to initiate the labeling. For the APEX2 peroxidase, we used the established effective labeling time of 1 min (Hung et al., 2016; Lam et al., 2015) to avoid prolonged exposure to toxic H_2_O_2_. To visualize biotinylated proteins, we used fluorescently labeled streptavidin. The cargo adaptor complex AP-1 orchestrates transport between the TGN and endosomes (Hirst and Robinson, 1998; Ren et al., 2013; Robinson, 2004) and is reported to predominantly localize to the TGN (Gravotta et al., 2012). Thus, we expected both the fusion protein and the pool of biotinylated proteins to localize in the TGN area. The different biotin ligases BioID2, miniTurboID and TurboID and the peroxidase APEX2 localize correctly when fused to endogenous AP1µA (Fig. 1D). Importantly, biotinylated proteins and AP1µA fusions are strongly concentrated to the TGN region, marked by the TGN-resident protein p230 (also known as GOLGA4) (Gleeson et al., 1996) (Fig. 1D). However, we could not find any specific biotinylation for the APEX2 peroxidase when expressed at physiological levels (Fig. 1D). To exclude general handling errors with the APEX2 sample, we transiently overexpressed vimentin–APEX2 as well as AP1µA–APEX2 fusions and found for both constructs a specific biotinylation pattern (Fig. S1A,B).

Quantitative analysis of the biotinylation rate of the BioID2, miniTurboID and TurboID KIs in immunofluorescence (Fig. S1C,D) showed that shorter (2 h) labeling with MiniTurboID and TurboID led to higher levels of biotinylated proteins than longer BioID2 labeling (24 h). Similar results were seen when levels of biotinylated proteins were probed on western blot (Fig. S1E). This correlates well to what was originally reported for those labeling enzymes (Branon et al., 2018; Kim et al., 2016). The low biotinylation rate of BioID2 makes it, in our opinion, unfavorable compared to the TurboID variants, as a high amount of biotinylation is crucial to enrich enough protein for the subsequent identification and quantification by MS. We decided to use the AP1µA^EN^–TurboID-V5 (EN represents endogenous) cell line for our further experiments as the TurboID fusion yielded a higher biotinylation rate compared to the other fusions (Fig. S1D).

### Physiological expression of TurboID fusions permits highly specific interactome mapping

**Fig. 2.**
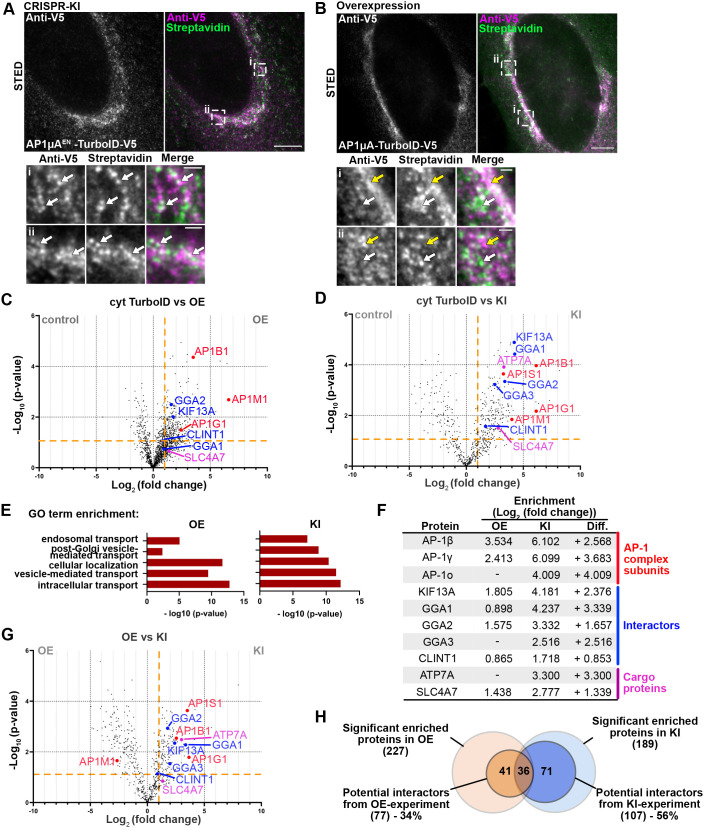
**Endogenous tagging allows for more specific proximity labeling and interactome mapping than overexpression of the labeling enzyme.** (A) STED micrographs of a fixed AP1µA^EN^–TurboID–V5 cell stained with anti-V5 antibody and streptavidin–STARORANGE to detect biotinylated proteins. Cells were treated with 50 µM biotin for 2 h before fixation. Magnified views show distinct overlap of biotinylated proteins and AP1µA^EN^-TurboID-V5 (marked by white arrows). (B) STED micrographs of a fixed cell transiently overexpressing AP1µA–TurboID–V5 that was treated as described in A. Magnified views show that biotinylated proteins and AP1µA–TurboID–V5 accumulate in distinct zones (white arrows indicate areas of biotinylation without AP1µA–TurboID–V5, yellow arrows indicate areas of accumulated biotin ligase without biotinylated proteins). Scale bars: 5 µm (main images); 500 nm (magnifications). Images in A and B representative of three repeats. (C) Volcano plot showing the changes in relative protein intensity between the overexpression (OE) experiment and control (cytosolic overexpressed TurboID). Significant hits are shown in the top right corner (*P*<0.05 and log_2_ fold change >1) separated by the orange lines. The volcano plot only includes proteins that were significantly enriched compared to WT cells. Subunits of the AP-1 complex (red), known interactors (blue) and known cargoes (magenta) are marked. The entire protein list is shown in Table S1. (D) Volcano plot showing the changes in relative protein intensity between the KI experiment (AP1µA^EN^–TurboID–V5) and control. Parameters are as in C. Data shown in the volcano plot is derived from three replicates. (E) GO term enrichment analysis showing enrichment of selected GO terms in the overexpression (OE) and KI condition. (F) Table of the analyzed subunits, interactors and cargo proteins. Differences (Diff.) in log_2_ fold enrichment are indicated. (G) Volcano plot showing the changes in relative protein intensity between KI experiment and OE experiment. Parameters are as in C. Data shown in the volcano plot is derived from three replicates. (H) Venn diagram showing the number of potential interactors [defined by protein localization and function (see Materials and Methods) and significant enrichment]. Lists of potential interactors are shown in Tables S2 and S3.

Transient overexpression of a protein can lead to artifacts, such as mislocalization or aggregation (Gibson et al., 2013; Moriya, 2015; Rizzo et al., 2009), increasing the chances of detecting non-specific, artificial interactors such as abundant cytosolic proteins. Physiological expression of the labeling enzyme should allow for highly specific, locally confined biotinylation of natural interactors. By applying two-color stimulated emission depletion (STED) super-resolution microscopy, we were able to visualize the biotinylation pattern after 2 h of biotin addition in AP1µA^EN^–TurboID–V5 KI cells (Fig. 2A) and compare it with AP1µA–TurboID–V5 overexpression (Fig. 2B). The high resolution achieved on the STED microscope enabled visualization of distinct nanodomains occupied by AP-1. Notably, the biotinylated proteins were primarily localized to the very same nanodomains as the endogenous AP1µA^EN^–TurboID–V5 fusion (indicated by the white arrows in Fig. 2A), indicating a high local specificity of the proximity labeling of the TurboID. Overall, localization of the overexpressed AP1µA–TurboID–V5 was more diffuse (Fig. 2B), and we observed areas where biotinylated proteins and fusion protein did not overlap (white and yellow arrows in Fig. 2B). Many cells overexpressing AP1µA–TurboID–V5 exhibited a very high background biotinylation in the cytoplasm and in the nucleus (Fig. S2A), whereas this was not noticed in the KI cells. Quantification of the background biotinylation revealed significantly higher cytosolic biotinylation in the transiently overexpressed cells compared to the KI cells, even after a short biotin incubation (Fig. S2B).

To probe whether endogenous tagging with TurboID allows biotin labeling of AP1µA-specific interactors with increased sensitivity compared to transient overexpression, we analyzed streptavidin-purified proteins by MS using label-free quantification. Biotin was added to the culture medium for 24 h to secure detectable protein labeling in the KI cells. We analyzed AP1µA^EN^–TurboID–V5 KI cells and cells transiently overexpressing an AP1µA–TurboID–V5 fusion protein. As controls we used cells transiently overexpressing a cytosolic TurboID–V5 as well as wild-type (WT) HeLa cells that were treated with biotin for 24 h. In total, we identified and quantified 4548 proteins (Table S1). Proteins with at least a 2-fold increase in relative intensity compared to both controls (log_2_ fold change >1) and a *P*<0.05 were considered significantly enriched. We found 227 proteins significantly enriched in the overexpression sample and 189 proteins significantly enriched in the KI sample (Fig. 2C,D). Overall, a larger number of proteins were enriched when AP1µA–TurboID was overexpressed compared to the KI condition, which is likely to be the result of mislocalization of AP1µA–TurboID–V5, possibly leading to the biotinylation of a larger set of proteins that naturally would not interact with AP-1. We found the two large subunits of the AP-1 complex (AP-1β1 and AP-1γ) (Hirst and Robinson, 1998) to be significantly enriched in overexpression condition (Fig. 2C) and even more enriched in the KI cells (Fig. 2D). Notably, the other small subunit of the AP-1 complex (AP-1σ) (Hirst and Robinson, 1998) was only identified when AP1µA^EN^–TurboID–V5 was endogenously expressed. We next looked at known interactors of the AP-1 complex to test whether endogenous tagging improves enrichment of specific interactors. The AP-1 complex is recruited to the Golgi membranes where it directly binds to the clathrin adaptors EpsinR (also known as CLINT1) and Golgi-localized, γ-ear-containing, Arf-binding proteins (GGAs) (Doray et al., 2002; Mills et al., 2003). Cell motility of membrane-bound AP-1 is conferred through the kinesin-like protein KIF13A, a microtubule-dependent motor protein that directly interacts with AP-1 (Nakagawa et al., 2000). Most interactors were identified in the overexpression condition, but only GGA2 and KIF13A were found to be significantly enriched. Physiological expression of AP1µA^EN^–TurboID–V5, by contrast, allowed significant enrichment of KIF13A, EpsinR and all GGA proteins (GGA1–GGA3). The difference between physiological expression levels and overexpression was even more striking when we looked at the AP-1-specific cargo proteins ATP7A and the sodium-bicarbonate co-transporter NBCn1 (SLC4A7) (Holloway et al., 2013; Severin et al., 2023). These AP-1 cargo proteins were all not or only slightly enriched in the overexpression experiment but significantly enriched in KI cells.

The overall impression that physiological expression of the TurboID fusion enhances the specificity of the hits we derive from our interactome data compared to overexpression can be confirmed when looking at gene ontology (GO) term analysis. Although both datasets show enrichment of general GO terms, such as ‘intracellular transport’ or ‘vesicle-mediated transport’, AP-1-specific GO terms including ‘post-Golgi vesicle-mediated transport’ or ‘endosomal transport’, are more frequent in the KI sample (Fig. 2E). Likewise, direct comparison between the two datasets shows significantly higher enrichment of all known interactors, subunits and cargo proteins in proximity labeling MS experiments performed in KI cells compared to what was seen with simple overexpression of the labeling enzyme (Fig. 2F,G). The exception here is AP1µA itself, as the overexpression of the fusion proteins led to higher enrichment in the overexpression (OE) condition (Fig. 2G).

To finally evaluate the overall quality of the MS data from the CRISPR-KI AP1µA^EN^–TurboID experiment, we identified possible interactors of AP1µA from the MS data and compared the two datasets (KI versus OE). We defined potential interactors as proteins that were significantly enriched and are known to localize to the either the Golgi or the TGN, are involved in cellular trafficking, are transmembrane proteins that might be trafficked by AP1µA or might be involved in the regulation of membrane homeostasis (e.g. regulatory kinases). For the KI cell line, we found a total of 107 potential interactors (Table S2), which corresponds to 56% of all significant enriched hits in the MS. Evaluation of the MS data from the overexpressed AP1µA–TurboID resulted in a list of only 77 potential interactors (34% of total number of significant hits) (Table S3). The results of our analysis are illustrated in Fig. 2H. Taken together, our findings highlight the importance of physiological expression levels of the TurboID fusion protein. By tagging the AP1µA endogenously, we increased the sensitivity of the proximity biotinylation and the MS measurement so that very transient but specific interactors, such as specific cargo proteins, could be identified.

### Interactome mapping of different AP complexes with endogenous TurboID fusions

The comprehensive interactome data resulting from endogenous tagging of AP1µA with TurboID encouraged us to apply the rapid KI TurboID approach to different proteins to probe its versatility and, in particular, to reveal the native interactome of AP complexes. Five different AP complexes (AP-1, AP-2, AP-3 and AP-4 and the more recently discovered AP-5) are responsible for sorting cargo throughout the endo-lysosomal system of human cells (Sanger et al., 2019). Our overall understanding of the intracellular role of the different AP complexes would greatly benefit from a better knowledge of their interactome. Aside from a few cargo proteins that are often used as model cargoes, little is known about which proteins are sorted by which adaptor protein in mammalian cells (Tan and Gleeson, 2019). Recent proteomic studies have shed light on cargo sorting pathways in yeast cells (Eising et al., 2022); however, such a comparative and comprehensive study is still lacking for mammalian cells. We also do not fully understand the mechanisms of recruitment of AP complexes to different membranes. Although AP-1, AP-3 and AP-4 are all known to be recruited by ARF1 to the TGN or endosomal membranes (Park and Guo, 2014), they localize to distinct endosomal buds (Peden et al., 2004; Theos et al., 2005), suggesting the presence of different unknown interactors. Owing to their role as key regulators of intracellular trafficking, dysfunction of AP complexes is linked to a variety of diseases (Park and Guo, 2014; Sanger et al., 2019; Shin et al., 2021) and a better understanding of the interaction partners could help reveal the underlying mechanistic basis of pathology. AP-1 is known to locate to the TGN and endosomal membranes (Peden et al., 2004; Ren et al., 2013; Wang et al., 2003), where it coordinates clathrin-dependent trafficking between the two organelles (Hirst and Robinson, 1998; Kirchhausen et al., 2014; Waguri et al., 2002). AP-2 is found at the plasma membrane, where it recruits clathrin for clathrin-mediated endocytosis (Hirst and Robinson, 1998). AP-3 is thought to localize to the same endosomes as AP-1 but has different cargo clients, which points towards a role in trafficking to the lysosome (Ihrke et al., 2004; Peden et al., 2004). AP-4 binds to TGN membranes (Sanger et al., 2019), where it mediates transport of autophagosomal factor ATG9A (Davies et al., 2018; Mattera et al., 2017). The low abundance of AP-4 [∼40-fold lower than AP-1 or AP-2 in HeLa cells (Itzhak et al., 2016)] makes it an interesting target to test the endogenous TurboID tagging on a very low abundant protein. AP-5 is thought to be involved in the transport from the late endosome to the Golgi or the lysosome and to also contribute to lysosome maintenance (Hirst et al., 2021, 2018). It is different from other AP complexes as it associates with two additional proteins (Hirst et al., 2011) and is not recruited to intracellular membranes by ARF1 as are AP-1, AP-3 and AP-4. However, as it has a low abundance comparable to that of AP-4 (Hirst et al., 2018) and has not been precisely localized so far, we decided not to include it in this study as it would be difficult to confirm correct integration of the endogenously tagged subunit.

**Fig. 3.**
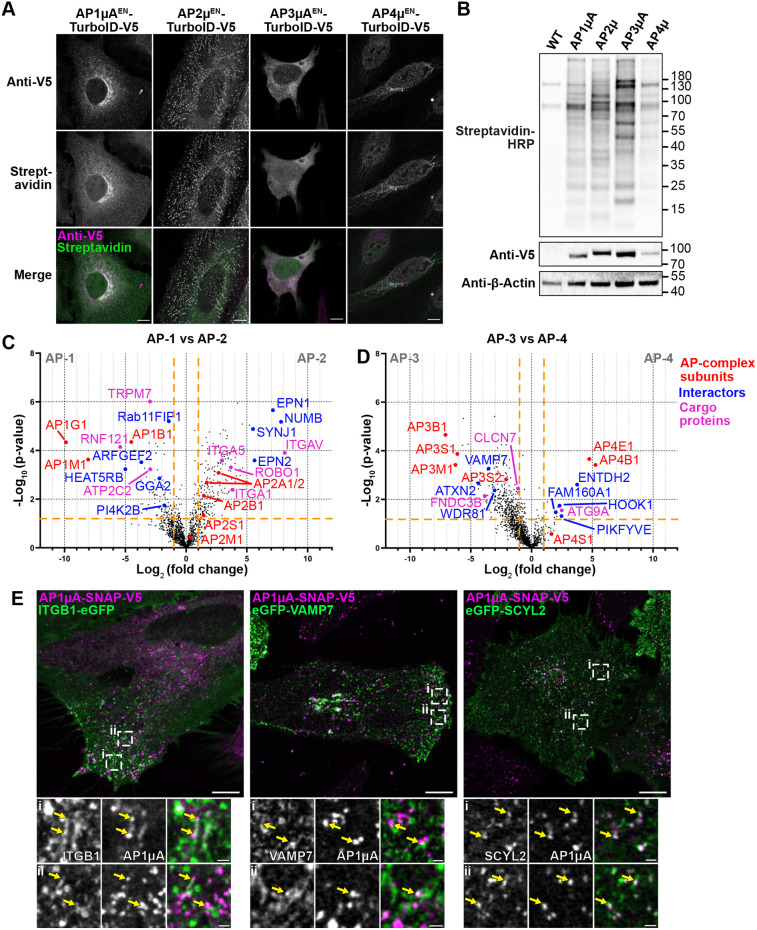
**Interactome analysis of AP complexes with endogenous TurboID tagging.** (A) HeLa KI cells expressing the endogenous AP µ subunits 1–4 fused to TurboID–V5 were fixed and stained with anti-V5 antibody to detect the labeling enzyme and streptavidin–AF488 to detect biotinylated proteins. Cells were incubated with biotin (50 µM) for 24 h before fixation. (B) Visualization of TurboID activity in all four KI cell lines on a western blot. Cells were treated with 50 µM biotin for 24 h. Whole-cell lysates were blotted with streptavidin–HRP to detect biotinylated proteins, and anti-V5 antibody to detect ligase expression. (C) Volcano plot showing the changes in relative protein intensity between AP1µA^EN^–TurboID–V5 and AP2µ^EN^–TurboID–V5. Proteins that show significant changes in their relative intensity are shown in the top left (AP-1) and top right (AP-2) corner (*P*<0.05 and log_2_ fold change >1 or <−1) separated by the orange lines. Subunits of the AP complexes (red), potential interactors (blue) and potential cargoes (magenta) are marked. (D) Volcano plot showing the changes in relative protein intensity between AP3µA^EN^–TurboID–V5 and AP4µ^EN^–TurboID–V5. Parameters are as in C. (E) HeLa AP1µA^EN^–SNAP–V5 KI cells labeled with JFX_650_-BG (BG, benzylguanine) that were transiently transfected with plasmids encoding for ITGB1–eGFP, eGFP–VAMP7 and eGFP–SCYL2 (left to right). Magnifications show where AP1µA domains are observed in close proximity to structures defined by the various proteins tested (marked by yellow arrows). All images representative of three repeats. Data shown in volcano plots in C and D is derived from four replicates. Scale bars: 10 µm (A; E, main images); 1 µm (E, magnifications).

We C-terminally tagged the µ-subunit of the different AP complexes with TurboID and tested the expression and localization of the fusion protein with immunofluorescence imaging and western blotting (Fig. 3A,B). After 24 h of biotin treatment, we were able to detect extensive biotinylation of proteins via western blotting and in immunofluorescence imaging experiments for all AP complexes, even for the low abundance AP4µ^EN^–TurboID–V5 fusion. To map and compare the interactome of the four AP complexes, we analyzed streptavidin-purified biotinylated proteins by MS using label-free quantification for all the KIs. As a control, we used HeLa WT cells that were treated with biotin. In total, we identified and quantified 2574 proteins (Table S4). In order to identify specific interactors and cargo proteins for each AP complex, we compared the relative intensity of a given protein between datasets for various AP complexes. Proteins with at least a 2-fold increase in relative intensity (log_2_ fold change >1 or <-1) and a *P*-value <0.05 were considered significantly enriched. By doing so, we were able to identify AP-2 complex-specific interactors such as epsin-1 (EPN1) and epsin-2 (EPN2), synaptojanin-1 (SYNJ1) and the protein numb homolog (NUMB) that are all known for their role in clathrin-mediated endocytosis (Fig. 3C). Importantly, the dataset for the low abundance AP4µ^EN^–TurboID–V5, highlighted several known AP-4 specific interactors such as tepsin (ENTDH2), HOOK1 and a FHF complex subunit (FAM160A1, encoded by *FHIP1A*) (Mattera et al., 2020) (Fig. 3D). Comparison of the interactome of various AP complexes allowed not only the identification of interactors that are specific for one single AP complex [e.g. GGA proteins and the PI4-kinase PI4K2B for AP-1 (Bai et al., 2004; Wieffer et al., 2013)], but also revealed potential common interactors, such as the SNARE protein Vamp7, which is enriched for AP-1 as well as AP-3 (Fig. 3D). Aside from potential uncharacterized effectors, endogenous TurboID tagging enabled the identification of potential cargo proteins specific for each AP complex. We identified known cargo proteins [various integrins (ITGA5, ITGAV or ITGA1) for AP-2 or ATG9A for AP-4] and also novel potential cargo proteins, such as the ring finger protein 121 (RNF121), a Golgi-localized protein with anti-apoptotic effects in cancer cells (Zhao et al., 2014), the plasma-membrane-localized cation channel TRPM7, the TGN-localized Ca^2+^ transporter ATP2C2 for AP-1 and the lysosomal Cl^−^/H^+^ Antiporter ClC-7 (CLCN7) for AP-3 (Fig. 3C,D). Those potential cargo proteins all have at least one tyrosine-based YXXϕ (ϕ represents a hydrophobic residue) motif in their cytoplasmic domains that is one of the motifs necessary for sorting via AP-1 or AP-3. We compared the significantly enriched proteins from the datasets of each AP complex and assembled a list of potential interactors and potential cargo proteins for each AP complex (Table S5). Potential interactors and cargo proteins were defined proteins that are known to be involved in membrane trafficking, might be involved in regulation of membrane homeostasis (e.g. regulatory phosphatases and kinases), have a transmembrane domain (potential cargo proteins) or are known to play a role in clathrin mediated endocytosis (for AP-2). In total, we identified more than 300 potential interactors and more than 200 potential cargo proteins. We think that proteins that are enriched for multiple APs are particularly interesting candidates for future research.

We then further tested some of the unexpected hits we found in the AP1µA data. We selected integrin β1 (ITGB1), the SNARE protein VAMP7 and SCY1-like 2 (SCYL2). ITGB1 could be a potential cargo protein of AP-1 (Kell et al., 2020). VAMP7 is a component of a SNARE complex composed of syntaxin-8, syntaxin-7 VAMP7 and VTI1B that is involved in endosomal recycling of endocytosed material (Bogdanovic et al., 2002), and, interestingly, we found all four members as potential AP-1 interactors. So far, VAMP7 has only been reported to interact with AP–3, and no direct interaction with the other adaptor complex, AP-1, has been observed (Kent et al., 2012; Salazar et al., 2006). SCYL2 was originally identified as a protein kinase for AP-2 (Conner and Schmid, 2005) but has also been connected to AP-1- and AP-3-mediated trafficking (Düwel and Ungewickell, 2006), as well as clathrin-dependent TGN export in plants (Jung et al., 2017), but its overall role remains poorly understood. We transiently expressed GFP fusions of the three proteins in an AP1µA^EN^–SNAP–V5 KI cell line, where AP1µA has been tagged with a SNAP tag (Fig. S3), enabling visualization of endogenous AP1µA in living cells. Live-cell confocal microscopy showed that vesicular AP-1 structures can be found on tubular compartments positive for both ITGB1 and VAMP7 (Fig. 3E). Similarly, we find punctuated SCYL2 structures perfectly colocalizing with vesicular AP1µA (Fig. 3E). All three proteins are thus likely to interact with the adaptor complex AP-1, as suggested by their close proximity in living cells. Interestingly, ITGB1 is found together with AP-1 in the peripheral areas of the cell, pointing towards a possible role for AP-1 in the endocytic recycling of the integrin, a role that has been described for AP-1 in the recycling of the transferrin receptor (Hirst et al., 2005). The close proximity of SCYL2 and VAMP7 to vesicular AP-1-positive structures suggests some regulatory functions for those proteins in AP–1 mediated trafficking.

### N-terminal endogenous tagging to map the interactome of CLCa

**Fig. 4.**
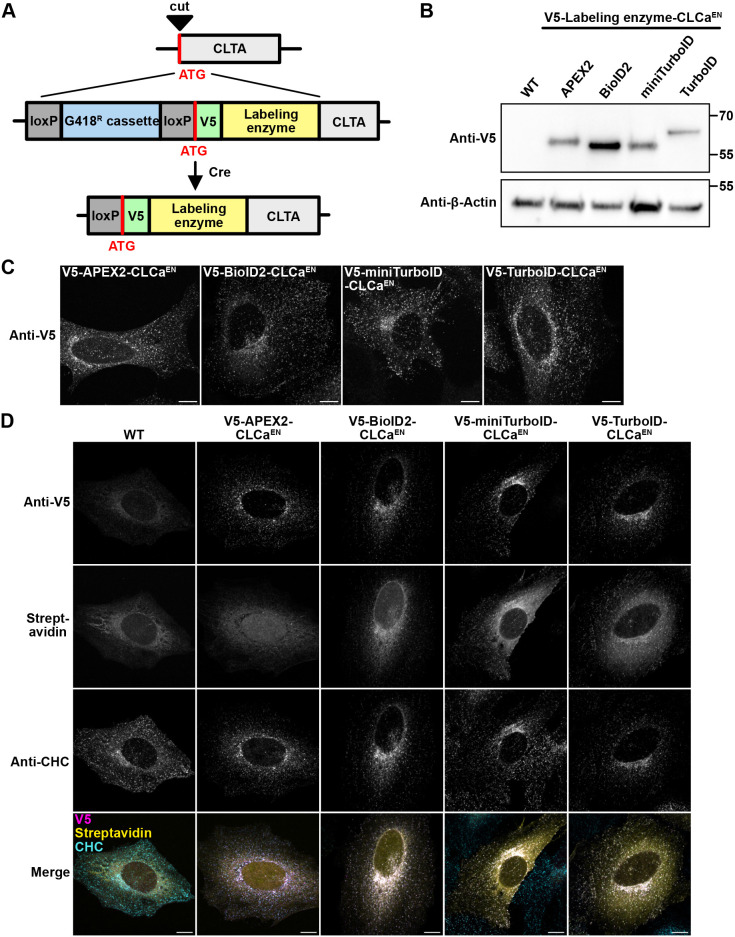
**Endogenous N-terminal tagging of CLCa with labeling enzymes.** (A) Scheme of the KI strategy. CLCa was N-terminally tagged with the labeling enzyme and a V5 tag. The integrated resistance cassette can be excised after transfection with the Cre recombinase. (B) Blots of whole-cell lysates from generated cell lines to verify the KI of the labeling enzymes by anti-V5 blotting. (C) Cell lines expressing the labeling enzymes endogenously were fixed and stained for the V5 tag to detect the labeling enzyme expression. (D) Cells endogenously expressing the different labeling enzymes were fixed and stained with an anti-V5 antibody to detect the labeling enzymes, anti-CHC antibody to detect the endogenous clathrin heavy chain and streptavidin–AF488 to detect biotinylated proteins. Cells were treated with 50 μM biotin for 24 h and V5-APEX2-CLCa^EN^ expressing cells were incubated for 30 min with 500 μM biotin-phenol and labeling was induced for 1 min with H_2_O_2_. All images representative of three repeats. Scale bars: 10 µm.

To test whether we can also apply our rapid KI strategy for N-terminally tagged proteins, we fused the different labeling enzymes to the N-terminus of endogenous clathrin light chain A (CLCa). To create N-terminal fusions, the resistance cassette was inserted between LoxP sites upstream of the start codon of the V5 tag and the TurboID (Schmidt et al., 2016). In a second step, the resistance cassette was then excised via transfection of Cre recombinase. This step becomes necessary as the presence of the resistance cassette in the genome could possibly isolate the gene from its promoter region and prevent its transcription. The strategy used for N-terminal tagging is illustrated in Fig. 4A. Successful expression of the fusion protein was confirmed by western blotting and with immunofluorescence microscopy (Fig. 4B,C). Moreover, immunofluorescence microscopy of the CLCa fusion proteins together with clathrin heavy chain (CHC) shows that both proteins colocalize, suggesting correct localization and function of the fusion proteins (Fig. 4D). The percentages of cells expressing the fusion proteins were comparable to those seen with C-terminal tagging [50% (APEX2), 45% (BioID2), 46% (MiniTurboID), 56% (TurboID)]. As for C-terminally tagged AP1µA, we observed locally confined biotinylation for the biotin ligases but no specific biotinylation for the APEX2 fusion protein (Fig. 4D).

**Fig. 5.**
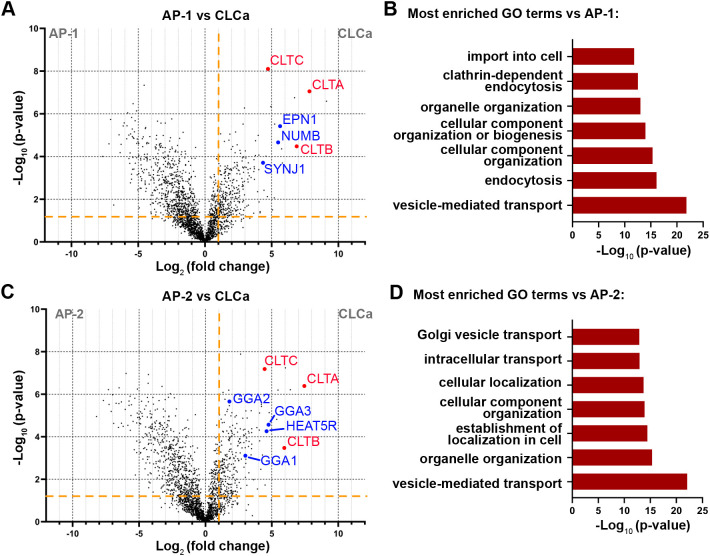
**Comparison of interactome datasets allows mapping of pathway specific clathrin interactors.** (A) Volcano plot showing the changes in relative protein intensity between AP1µA^EN^–TurboID–V5 and V5–TurboID–CLCa^EN^. Significant hits (*P*<0.05 and log_2_ fold change >1) are separated by the orange lines in the top right. Clathrin chains (red) and example proteins involved in clathrin-mediated endocytosis (blue) are marked. (B) GO term enrichment analysis showing the most enriched GO terms comparing enrichment for CLCa against AP1µA. (C) Volcano plot showing the changes in relative protein intensity between AP2µ^EN^–TurboID–V5 and V5–TurboID–CLCa^EN^. Parameters are as in A. Clathrin chains (red) and example proteins involved in post-Golgi transport (blue) are marked. (D) GO term enrichment analysis showing the most enriched GO terms comparing enrichment for CLCa against AP2µ. Data for volcano plots and GO term enrichment diagrams is derived from four replicates.

We then used the V5–TurboID–CLCa^EN^ KI cell line to map the CLCa interactome by analyzing streptavidin-purified biotinylated proteins with MS using label-free quantification (Table S6). CLCa fulfils multiple roles in intracellular trafficking, such as coating membranes during endocytic events or trafficking intermediates shuttling between the Golgi or TGN and endosomes (Kirchhausen, 2000). Clathrin does not directly bind membranes but uses adaptor proteins, such as adaptor protein complexes AP-1, for Golgi-endosome trafficking and AP-2 for clathrin-mediated endocytosis (Hirst and Robinson, 1998). As we already have performed interactome mapping experiments for those two adaptors, a direct comparison between the datasets generated from CLCa and AP-1 or AP-2 TurboID KIs allowed separation of CLCa interactors that are involved in endocytosis from those involved in intracellular post-Golgi transport. A comparison of CLCa against AP-1 shows strong enrichment of proteins such as EPN1, SYNJ1 and NUMB, which are known for their role in clathrin-mediated endocytosis (Fig. 5A). Furthermore, GO terms such as ‘endocytosis’, ‘clathrin-mediated endocytosis’ and ‘import into cell’ are strongly enriched (Fig. 5B). By contrast, comparison of the interactome of CLCa with AP-2 highlighted proteins that are involved in AP-1-dependent post-Golgi transport, such as the GGA proteins and the HEAT repeat containing 5B (HEATR5B) (Hirst et al., 2005) (Fig. 5C). Here, among the most enriched GO terms we find ‘intracellular transport’ and ‘Golgi-vesicle transport’ (Fig. 5D). In conclusion, endogenous N-terminal tagging of CLCa with TurboID granted a highly specific interactome dataset that, when combined with datasets from AP-1 or AP-2, allowed mapping of pathway-specific interactors.

## DISCUSSION

Employing an antibiotic-based CRISPR KI strategy for C- and N-terminal tagging, we endogenously fused the commonly used labeling enzymes APEX2, BioID2, miniTurboID and TurboID to AP1µA and CLCa (Figs 1, 4). We identified the biotin ligase TurboID as best suited for the KI approach in combination with proximity labeling experiments, as it exhibits favorable labeling kinetics (Fig. S1). The lower number of cells expressing miniTurboID compared to TurboID might be result of lower stability of miniTurboID fusion proteins, which has been previously reported in other studies (Branon et al., 2018; May et al., 2020). Faster labeling kinetics are not only beneficial to reliably generate sufficient amounts of biotinylation to enrich enough protein for the subsequent identification and quantification by MS but are also advantageous when designing experiments that require short labeling times. Generally, the incubation time with the biotin might be adapted according to the abundance of the POI; when working with highly abundant proteins, incubation times shorter than 24 h with biotin could be used. Previous work has already demonstrated the TurboID variants outperform BioID or BioID2 regarding their labeling kinetics (Branon et al., 2018; Cho et al., 2020; May et al., 2020; Zhang et al., 2019); however, a larger effective labeling radius might lead to less specific datasets (May et al., 2020). Recently, new variants of the BioID2 have been introduced, named microID and ultraID (Kubitz et al., 2022). With a molecular mass below 20 kDa, they are significantly smaller than the here presented labeling enzymes. Especially ultraID is reported to have labeling kinetics similar to TurboID with less background activity. In addition, an ancestral BioID variant called AirID has been developed with faster labeling kinetics than BioID and more specific labeling compared to TurboID (Kido et al., 2020). Another possibility to reduce labeling background of TurboID is the use of a light-controlled variant of the TurboID (LOV-Turbo) (Lee et al., 2023). It would be interesting to see how these variants perform when used for tagging at the endogenous locus. Practically, labeling with heavy biotin and detection of heavy biotin-modified peptides could be a solution to distinguish background biotinylation from induced biotin-labeling in TurboID experiments. This becomes crucial in interactome mapping experiments that, for example, require induced changes of the cell state, as labeling should only occur after the change was induced. It needs to be noted that TurboID and especially miniTurboID protein fusions are reported to be less stable than BioID and might cause cell toxicity (May et al., 2020). When probing expression of the fusion proteins on western blots, we indeed noticed that BioID2 fusions are expressed at higher levels than miniTurboID, possibly due to higher stability of the fusion protein. We did not observe any signs of induced toxicity in our KI cells, as cell growth and cell shape were comparable between KI cell lines and WT cells. However, this has to be tested for each protein tagged.

To our surprise, we could not achieve any biotinylation with the APEX2 peroxidase expressed as a low abundance endogenous fusion (Figs 1, 4). The short labeling time in combination with the low physiological expression did not yield detectable proximity labeling in our hands. Generally, CRISPR KI approaches to tag endogenously with APEX2 have been reported as functional (Gupta et al., 2018; Markmiller et al., 2018); however, for us, multiple attempts to induce labeling failed. We can only speculate that highly concentrated APEX2 is needed to get sufficient biotinylation when it is endogenously expressed. Specific confined cellular environments, such as nucleus or stress granules, would favor such a high concentration of an APEX2 fusion protein. On the other hand, soluble cytosolic proteins might not be optimal for an APEX2 approach. However, labeling with TurboID and biotin worked reliably and was easy to induce for peripheral cytoplasmic machinery like adaptors and clathrin.

Using the AP1µA^EN^–TurboID–V5 cell line created with the KI strategy, we were able to compare the enrichment of known interactors and cargo proteins of the AP-1 complex in quantitative MS measurements against datasets generated with cells overexpressing AP1µA–TurboID–V5. Strikingly, we found known interactors to be significantly more enriched when AP1µA^EN^–TurboID–V5 was expressed at endogenous levels (Fig. 2). Especially relevant for our research is the strong enrichment of AP-1 cargo proteins that can be only observed when TurboID is endogenously fused to AP1µA, highlighting the importance of matching the endogenous expression levels. The increased sensitivity for real interactors is likely a result of less unspecific labeling due to mislocalization or aggregation artefacts induced by overexpression of the labeling enzyme fused to the POI. A larger background of peptides from unspecifically labeled proteins increases the sample complexity and therefore lowers the overall sensitivity of the MS measurements, as all peptides compete for ionization and detection. Endogenous protein tagging with a biotin ligase allows for highly confined biotinylation (Fig. 2A) and therefore increases the chances of detection of specific interactors, especially if they are of low abundance. Importantly, endogenous expression of the AP1µA^EN^–TurboID fusion resulted in a more comprehensive list of potential interaction partners compared to overexpression of AP1µA–TurboID (Tables S2, S3; Fig. 2E,H). Using the described KI pipeline, we tagged the µ-subunit of different AP complexes with TurboID and comparably analyzed their interactome in quantitative MS measurements (Fig. 3). The provided lists of potential interactors and cargo proteins for each individual AP complex (Table S5) not only demonstrate the versatility of the approach but also present a database that can contribute to the better understanding of AP–driven intracellular sorting. For AP-4, different approaches have been compared to identify AP–4 interactors (Davies et al., 2018). Our results are comparable to data generated using an AP4ε–BioID fusion protein. Furthermore, AP-4 cargoes could only be identified in a sensitive immunoprecipitation experiment using the AP-4 interactor tepsin. This highlights general limitations of biotin-based proximity labeling approaches, as cargoes without or with only short cytosolic tails might not have lysine residues as potential acceptors for biotinylation, and cargoes which are luminal and are transported in vesicular or tubular transport intermediates are not accessible for biotinylation. For identification of such proteins, alternative approaches such as immunoprecipitation, cross-linking MS or immunoisolation of whole carriers or compartments would be required.

We believe that our datasets can be used as a starting point for studies aimed at unravelling the mechanisms of spatially confined recruitment of different TGN- and endosome-associated AP complexes. Furthermore, linking the AP complexes to the different potential cargo proteins will shine light on the intracellular sorting and trafficking routes of those proteins. For re-evaluation, all raw data have been made available via ProteomeXchange with identifier PXD051393. By picking three proteins from our list of potential AP-1 interactors (ITGB1, VAMP7 and SCYL2) and studying their localization in respect to AP-1 in living cells (Fig. 3E), we could show that they all localize in the vicinity of AP-1. Finally, we mapped the interactome of the N-terminally TurboID-tagged CLCa and compared the data to the datasets of AP1µA and AP2µ (Fig. 5, Table S6). By doing so, we were able to identify pathway-specific interactors of CLCa.

In summary, endogenous tagging with biotin ligases enables highly specific proximity labeling and increases the sensitivity for real interactors that might be transient or of low abundance. Our pipeline presents an alternative to classical CRISPR-based approaches that would require single-cell selection of positive TurboID clones and can be applied to any cell line as long as it can be reliably transfected and selected. The pipeline for the generation of CRISPR KIs, based on the integration of a resistance cassette, allows rapid generation of endogenously tagged cells and can be applied to low abundant target proteins like AP-4. Aside from obvious time and work reduction, it also avoids artefacts that arise from single clone behavior. In particular, for naturally low abundant proteins, inducible systems might still trigger non-physiological protein expression. Nevertheless, expression levels of the edited protein should always be controlled when applying the presented strategy for C-terminal KIs, as the 3′-UTR is shortened and might lose important gene-specific regulatory elements. When expression levels are affected, the loxP-based strategy could be applied also for C-terminal KIs, to remove the resistance cassette after selection.

Rapid endogenous tagging with TurboID enabled us to map and compare the interactome of four different AP complexes, which revealed known as well as novel AP-specific interactors. The ease and speed of the KI generation makes it an attractive alternative to transient overexpression of the labeling enzyme or classical KI approaches using single-cell selection, as the MS experiments can be performed in ∼4–5 weeks after the CRISPR transfection. Additionally, the tools provided here can be used in a variety of different cell lines for the identification of cell type-specific and physiological cargoes.

## MATERIALS AND METHODS

### Antibodies and streptavidin conjugates

All the streptavidin conjugates and antibodies used in this study are provided in Table S7.

### Mammalian cell culture

All experiments were carried out in HeLa cells ATCC CCL-2 (from the ECACC general collection) grown in a humidified incubator at 37°C with 5% CO_2_ in DMEM (Gibco, Thermo Fisher Scientific) supplemented with 10% fetal bovine serum (Corning) and penicillin-streptomycin (Lonza Bioscience) to prevent contamination. For transient transfection, HeLa cells at 70–80% confluency were transfected with FuGENE HD Transfection Reagent (Promega) according to the supplier's protocol.

### Generation of CRISPR-Cas9 knock-in cell lines

All primers used in this section can be found in Table S8.

The C-terminally tagged AP complex cell lines were generated following the strategy presented in Fig. 1A. The AP1M1 genomic locus (Gene ID 8907) was targeted shortly after the stop codon with the following guide RNA: 5′-CAGCCAACACCCCGGCCTCGGGG-3′ (PAM site underlined). The guide RNA was cloned into the SpCas9 pX459 plasmid (Addgene plasmid #62988) (Ran et al., 2013) by annealing oligonucleotides and ligation into the vector which was linearized with BbsI. The homology repair (HR) plasmid to generate the AP1µA-labeling enzyme cell lines contained ∼1 kb homology arms and was synthesized by Twist Bioscience. A glycin-serin linker (GSGSGSGSGS) and a BamHI and EcoRI site were designed between the two homology arms for the cloning of tags and resistance cassette as indicated in the schematic in Fig. 1A. The coding sequences of the different labeling enzymes were integrated between the homology arms, followed by a polyA sequence and a G418 resistance cassette that allows selection of positive edited cells with the drug G418/Geneticin (Thermo Fisher Scientific). The coding sequences of the various labeling enzymes were obtained via PCR from previously described vectors (Addgene plasmids #66170, #74224, #107171, #107172) (Branon et al., 2018; Kim et al., 2016; Lam et al., 2015) using sense primers with a BamHI restriction site and antisense primers with a NheI restriction site. The coding sequence for the SNAP tag was obtained from pSNAPf vector (New England Biolabs) via PCR using a sense primer with a BamHI restriction site and an antisense primer with a NheI restriction site. The SV40 polyA sequence was amplified from pEGFP-C1 (Clontech) using a PolyA NheI sense and a PolyA NotI antisense primers. The G418 resistance cassette was amplified from pEGFP-C1 using a G418 NotI sense and G418 EcoRI antisense primer. The various fragments were cloned into the HR vector linearized with BamHI and EcoRI. HeLa cells were transfected with 1 µg of pX459 plasmid with the AP1µA guide and 1 µg of HR-plasmid using FuGENE. G418 was added to the cells 3 days after transfection at a concentration of 1.5 mg/ml and medium was exchanged every 2–3 days with new G418 at the same concentration until the selection was complete (after 7–10 days). After selection the cells were passaged every 2-3 days with a 0.5 mg/ml maintenance concentration of G418.

The AP2µ^EN^–TurboID–V5, AP3µA^EN^–TurboID–V5 and the AP4µ^EN^–TurboID–V5 cell lines were generated in a similar way to the AP1µA^EN^–TurboID–V5 cell line.

The genomic locus of AP2M1 (Gene ID 1173) was targeted shortly after the stop coding with the guide RNA: 5′-ACTCGCTGCTAGCTGCCACTAGG-3′ (PAM site underlined). The guide RNA was cloned into the SpCas9 pX459 plasmid as described for AP1µA. The HR plasmid was synthetized by Twist Bioscience (∼1 kb homology arms). As for the AP1µA HR plasmid, a glycin-serin linker and a BamHI and EcoRI site were designed between the two homology arms. To generate the AP2µ^EN^-TurboID-V5-PolyA-G418 HR plasmid the entire insert (TurboID–V5–PolyA–G418) was excised from the AP1µA^EN^-TurboID-V5-PolyA-G418 HR plasmid using the BamHI and the EcoRI site. The insert was cloned into the ordered AP2µ HR vector linearized with BamHI and EcoRI. The AP2µ^EN^–TurboID–V5–PolyA–G418 KI cell line was generated as described for AP1µA using the AP2µ guide vector and the AP2µ-TurboID-V5-PolyA-G418 HR plasmid.

AP3µA^EN^–TurboID–V5 and AP4µ^EN^–TurboID–V5 cell lines were generated as described for AP2µ^EN^–TurboID–V5. The genomic locus of AP3M1 (Gene ID 26985) was targeted shortly after the stop coding with the guide RNA: 5′-TGGAAAACAAACTGGTCCTGAGG-3′ (PAM site underlined). The genomic locus of AP4M1 (Gene ID 9179) was targeted shortly after the stop coding with the guide RNA: 5′-GATCTGAGGCTCCCCAAACGAGG-3′ (PAM site underlined).

The AP4µ guide RNA was cloned into the SpCas9 pX330 plasmid (Addgene plasmid #42230) (Cong et al., 2013) by annealing oligonucleotides and ligation into the vector, which was linearized with BbsI.

The N-terminally tagged CLCa cells were generated following the strategy presented in Fig. 3A. The CLTA+genomic locus (Gene ID 1211) was targeted shortly after the start codon with the guide RNA: 5′-ATGGCTGAGCTGGATCCGTTCGG-3′ (PAM site underlined). The guide RNA was cloned into the SpCas9 pX330 plasmid by annealing oligonucleotides and ligation into the vector, which was linearized with BbsI. The CLCa HR plasmid was synthesized by Twist Bioscience. It contained both homology arms (∼1 kb), a short N-terminal glycin-serin linker (SGSGSGSG) and a V5 coding sequence with a start codon. NheI and BamHI sites were designed between left homology arm and V5 sequence to integrate the resistance cassette, and an EcoRI and SpeI site were designed for integration of the labeling enzymes. The coding sequences of the different labeling enzymes were obtained via PCR from previously described vectors (Addgene plasmids #66170, #74224, #107171, #107172) (Branon et al., 2018; Kim et al., 2016; Lam et al., 2015) using sense primers with an EcoRI restriction site and antisense primers with an SpeI restriction site. The G418 resistance cassette was amplified for pEGFP-C1 using a G418 NotI sense and G418 EcoRI antisense primer. A loxP sequence was integrated into both primers. The loxP-G418 fragment was cloned into the HR vector linearized with NheI and BamHI. In a second step, the labeling enzyme fragments were cloned into the HR vector with the G418 cassette linearized with EcoRI and SpeI. HeLa cells were transfected with 1 µg of pX330 plasmid with the CLCa-guide and 1 µg of HR-plasmid using FuGENE. After the selection with G418, the cells were again transfected with a CRE-transferase (Addgene plasmid #11923) (Le et al., 1999) using the FuGENE transfection agent.

### Plasmid design of overexpression plasmids

All primers used in this section can be found in Table S8.

The sequences encoding for AP1µA–APEX2–V5, AP1µA–TurboID–V5 and cytosolic TurboID–V5 were all cloned into the pEGFP-N1 plasmid. The fragments of the labeling enzymes were obtained via PCR from the used HR plasmids using sense primers with a BamHI restriction site and antisense primers with a NotI restriction site. The eGFP was removed from the pEGFP-N1 vector by digestion with BamHI and NotI and the labeling enzyme fragments were inserted. The cDNA sequence of AP1µA including the same GS linker that was used for the CRISPR KI was synthesized by Twist Bioscience and the AP1µA fragment was obtained via PCR using an EcoRI sense primer a BamHI antisense primer. The AP1µA fragment was inserted into the generated APEX2-V5 and TurboID-V5 vectors linearized with EcoRI and BamHI.

The Vimentin-APEX2-FLAG plasmid (Addgene #66170) (Lam et al., 2015) was used for transient expression of vimentin–APEX2. The pHcgreen ITGB1-GFP plasmid (Addgene #69804) (Parsons et al., 2008) was used for transient expression of ITGB1–eGFP. The pEGFP VAMP7 (1-220) plasmid (Addgene #42316) (Martinez-Arca et al., 2000) was used for transient expression of eGFP–VAMP7. For transient expression of eGFP–SCYL2, the coding sequence of SCYL2 was obtained from pDONR223-SCYL2 (Addgene #23458) (Johannessen et al., 2010) via PCR using a an EcoRI sense primer and an BamHI antisense primer. The SCYL2 fragment was cloned into the pEGFP-C1 vector using BamHI and EcoRI.

### Immunofluorescence

For all immunofluorescence samples, 40,000 cells were seeded on fibronectin-coated coverslips. Biotinylation was induced by replacing the growth medium with medium containing 50 µM biotin and samples were incubated for 24 h or 2 h as indicated in the figures. Biotinylation for AP1µA^EN^–APEX2–V5 cells was induced as described previously (Hung et al., 2016). All cells were washed twice with PBS and then fixed in 4% PFA for 10 min at room temperature (RT). Subsequently, they were rinsed three times with PBS and incubated for 3 min in permeabilization buffer [0.3% NP40 (Roth), 0.05% Triton-X 100 (Sigma Aldrich) and 0.1% BSA (IgG free) (Roth) in PBS]. Cells were blocked for 1 h in blocking buffer [0.05% NP40, 0.05% Triton-X 100 and 5% goat serum (Jackson ImmunoResearch) in PBS] at RT and then incubated with primary antibodies in blocking buffer overnight rocking at 4°C. On the next day, the samples were washed three times 5 min in washing buffer [0.05% NP40, 0.05% Triton-X 100 and 0.2% BSA (IgG free) in PBS] before incubation with the respective secondary antibodies in blocking buffer for 1 h rocking at RT. For visualization of biotinylated proteins either streptavidin–Alexa-Fluor-488 (AF488) (for confocal microscopy) or streptavidin–STARORANGE (for STED microscopy) was added to the secondary antibody mix. The cells were then washed three times 5 min with wash buffer and then dipped in dH_2_O before mounting with ProLong Gold Antifade Reagent (Thermo Fisher Scientific). Mounted samples were let to harden overnight at RT and then stored at 4°C until imaging.

### Live-cell imaging

For live-cell imaging experiments, 100,000 cells were seeded on glass-bottom dishes (3.5 cm diameter, no. 1.5 glass; Cellvis), coated with fibronectin (Sigma) beforehand. At 1 day after seeding, KI cells expressing the AP1µA–SNAP fusion were labeled using an O^6^-benzylguanine (BG) substrate [JFX650-BG, gift from the laboratory of Luke Lavis (NIH Janelia)] (1 µM) in culture medium for 1 h (Wong-Dilworth et al., 2023). After the labeling, cells were washed for at least 1 h in culture medium. Live-cell imaging was carried out in FluoroBrite DMEM (Gibco) supplemented with 10% FBS, 20 mM HEPES (Gibco) and GlutaMAX (Gibco). For live-cell imaging experiments a microscope incubator (Okolab) was used to keep the stage and sample at 37°C.

### Imaging and image processing

Confocal and STED imaging was carried out on a commercial expert line Abberior STED microscope equipped with 485 nm, 561 nm and 645 nm excitation lasers. For two-color confocal live-cell imaging, both signals were detected simultaneously, detection windows were set to 498 to 551 nm and 650 to 756 nm. For two-color STED experiments both dyes were depleted with a 775 nm depletion laser. The detection windows for the dyes were set to 498 to 551 nm, 571 to 630 nm and 650 to 756 nm. Excitation power was kept constant between samples in the same experiment to be able to quantify differences in expression levels and biotinylation. The pixel size was set to 60 nm for confocal and 20 nm for STED.

All images shown were smoothed using a Gaussian filter with 1-pixel s.d. using ImageJ (Abramoff et al., 2004). For better representation of the AP4µ^EN^-TurboID-V5 and live-cell images, the background was subtracted using a rolling ball radius of 50.0 pixels.

### Image analysis and statistical analysis

All image analysis was carried out with ImageJ. To determine the ratio of biotinylated proteins and V5-tagged AP1µA-labeling enzyme fusions in Fig. S1D, a small region in the Golgi area was selected in the raw image and the average grey values were measured for both channels. The ratio between the mean intensity fluorescence of the biotinylated proteins in the Golgi area versus the mean fluorescence intensities from the V5 channel was then calculated. For each condition, at least 30 cells were analyzed from three independent experiments.

To analyze background biotinylation in Fig. S2B, a small region in a cytoplasm was selected and the mean intensity fluorescence was measured. At least 20 cells from each condition were analyzed.

Statistical analysis (unpaired two-tailed *t*-tests) was carried out with Prism. *P*-values are indicated in figure legends.

### Quantification of the percentage of KI cells expressing the fusion proteins

To estimate the number of gene-edited cells after antibiotic selection that express the fusion protein, V5-immunostained cells for each KI cell line were screened for the presence of V5 signal using confocal light microscopy; 100 cells were screened for each cell line.

### KI verification via western blot

For each KI cell line, 180,000 cells were seeded on a 6-well plate. After 24 h, cells were washed twice with PBS and harvested in 350 µl of Laemmli sample buffer. The samples were boiled for 10 min at 98°C before loading 30 µl of each sample on two separate 4-12% SDS-PAGE gels (Thermo Scientific). After electrophoresis, proteins were transferred onto a nitrocellulose membrane (Amersham) via wet blotting. Both membranes were washed once with PBS with 0.05% Tween 20 (PBST) and then blocked for 1 h with 5% (w/v) milk powder and 1% BSA in PBST rocking at RT. After that, membranes were washed once 5 min with PBST and two times 5 min with PBS before incubation with the respective primary antibodies overnight rocking at 4°C. On the next day, the membranes were washed three times 5 min with PBST and incubated with a secondary antibody coupled to HRP in 5% (w/v) milk powder and 1% BSA in PBST for 30 min rocking at RT. The membranes were washed twice with PBST and twice with PBS for 5 min each. To develop the membrane, the ECL western blot substrate was added for 2 min and then the membrane was imaged.

Uncropped images of western blots are shown in Fig. S4.

### Western blot analysis of biotinylated proteins

For detection of biotinylated proteins on a western blot, 800,000 HeLa WT and all KI cell lines were seeded on a 10 cm cell culture dish. Starting the next day, the medium was replaced with biotin-containing medium (50 µM biotin) and samples were incubated for 24 h or 2 h at 37°C. The biotin addition was timed in a way that all samples could be harvested at the same time. Biotinylation for AP1µA^EN^–APEX2–V5 cells was induced as described previously (Hung et al., 2016). For harvesting, the cells were washed twice with ice-cold PBS and then extracted in 400 µl of ice-cold lysis buffer [50 mM Tris-HCl pH 7.4, 150 mM NaCl, 2 mM EDTA, 0.5% NP-40, 0.5 mM dithiothreitol (DTT) and protease inhibitors (Roche)]. Cells were then centrifuged for 10 min at 4°C at 14,000 ***g*** to clarify the cell lysates. 20 µl of the whole-cell lysates were mixed with Laemmli buffer and boiled at 95°C for 10 min before loading on a 4-12% SDS-PAGE gel. Two separate gels were used, one for detection of biotinylated proteins and the other for detection of the fusion protein. After electrophoresis, proteins were transferred to a nitrocellulose membrane via wet blotting. To visualize biotinylated proteins on the membrane, after blocking [5% (w/v) milk powder in PBST], the blot was incubated with 0.3 µg/ml streptavidin–HRP in 3% BSA in PBST for 30 min rocking at RT. The western blot to detect the fusion protein with the V5 tag was carried about as described above.

### Preparation of MS samples for the comparison of KI and overexpression

The protocol for MS sample preparation is based on the protocol described by Cho et al. (2020). In total, three independent samples for each condition were prepared. For each KI sample, AP1µA^EN^–TurboID–V5 cells were seeded into two T75 flasks (1.5 million cells per flask). For all other conditions (overexpression of AP1µA–TurboID–V5, cytosolic TurboID control and WT control), two 10 cm cell culture dishes were seeded with 1 million cells per dish. For transient overexpression of cytosolic TurboID or AP1µA–TurboID–V5, cells were transfected with 4 µg of either the AP1µA–TurboID–V5 pEGFP-N1 overexpression plasmid or the cytosolic TurboID–V5 pEGFP-N1 plasmid using FuGENE at 18 h after seeding. At 24 h after seeding, in all conditions, the medium was replaced with medium supplemented with 50 µM biotin. At 48 h after seeding the samples were washed five times with ice-cold PBS and then detached in 4 ml PBS per flask/10 cm dish using a cell scraper and collected in a falcon tube. Cells were pelleted by centrifugation at 300 ***g*** at 4°C for 3 min. The supernatant was removed and the pellet resuspended in 4 ml RIPA lysis buffer (50 mM Tris-HCl pH 7.4, 150 mM NaCl, 0.1% SDS, 0.5% sodium deoxycholate and 1% Triton X-100) supplemented with 1× protease inhibitors (Roche) and lysed for at least 10 min on ice. The cell lysates were distributed into microcentrifuge tubes then clarified by centrifugation at 13,000 ***g*** at 4°C for 10 min. The clarified lysates were then mixed with 200 µl of streptavidin magnetic beads that were previously equilibrated twice with RIPA lysis buffer. The samples were distributed into fresh microcentrifuge tubes and incubated with the magnetic beads rotating at 4°C overnight. On the next day, the beads were pooled and washed twice with RIPA lysis buffer (1 ml, 2 min), once with 1 M KCl (1 ml, 2 min) and then quickly once with 0.1 M Na_2_CO_3_ (1 ml, 10 s) and once with 2 M urea in 10 mM Tris-HCl (pH 8.0) (1 ml, 10 s). The beads were again washed twice in RIPA lysis buffer (1 ml, 2 min) and were then transferred into fresh microcentrifuge tubes. Subsequently, they were washed once with 50 mM Tris-HCl (pH 7.4) and twice with 2 M urea in 50 mM Tris-HCl (pH 7.4). The final wash buffer was then removed, and the beads were resuspended in 80 µl of 2 M urea in 50 mM Tris-HCl (pH 7.4) with 1 mM DTT and 0.4 µg trypsin and incubated for 1 h shaking at 1000 rpm. The supernatant was transferred into fresh microcentrifuge tubes and the beads were washed twice with 60 µl of 2 M urea in 50 mM Tris-HCl (pH 7.4). The washes were combined with the supernatant, DTT was added to a final concentration of 4 mM and the samples were incubated for 30 min at 25°C shaking at 1000 rpm. Next, iodoacetamide was added to final concentration of 10 mM and the samples were incubated in the dark at 25°C for 45 min while shaking at 1000 rpm. Finally, another 0.5 µg of trypsin were added and the digestion was proceeded overnight at 25°C shaking at 700 r.p.m. On the next day, the digestion was stopped by acidification with formic acid to a final concentration of 1% (v/v). The peptides were prepared for MS using SDB-stage tips.

### Preparation of MS samples for comparison of different AP complexes

For each sample (four independent samples in total), cells of the different KI cell lines (AP1µA^EN^–TurboID–V5, AP2µ^EN^–TurboID–V5, AP3µA^EN^–TurboID–V5 and AP4µA^EN^–TurboID–V5) were seeded into two 15 cm cell culture dishes (1.5 million cells per dish). Owing to the low abundance of AP-4, a third 15 cm cell culture dish with 1.5 million cells was used. As control, HeLa WT cells were seeded into two 15 cm cell culture dishes (1.5 million cells per dish). At 24 h after seeding, the medium was replaced with medium supplemented with 50 µM biotin for another 24 h. For harvesting and enrichment, the protocol was almost identical to the one described above. To ensure sufficient cell lysis and full disruption of membranes, all pellets were resuspended in 4 ml RIPA lysis buffer supplemented with 1× protease inhibitor and lysed for 30 min on ice. During this time, the cell membranes were disrupted by passing the cells 10 times (five strokes) through a 24 G needle.

### Preparation of MS samples for V5–TurboID–CLCa

For each sample (four independent samples in total), V5–TurboID-CLCa^EN^ cells were seeded into two 15 cm cell culture dishes (1.5 million cells per dish). As a control, HeLa WT cells were seeded in two 15 cm cell culture dishes (1.5 million cells per dish). At 24 h after seeding, the medium was replaced with medium supplemented with 50 µM biotin for another 24 h in all conditions. For harvesting and enrichment the protocol described above was used.

### Sample clean-up by SDB-STAGE tips

Salts and detergents were removed before liquid chromatography (LC)-MS analysis by SDB-RPS StageTips (Empore™ 2241). Stage tips were prepared as described previously (Rappsilber et al., 2007). For clean-up, StageTips were conditioned with 200 μl of 100% methanol, 200 μl of solvent 1 (80% acetonitrile, 0.1% formic acid), and twice with 200 μl of solvent 2 (0.1% formic acid). Then, acidified peptides were loaded onto the conditioned StageTips and the StageTips were washed twice with 200 μl of solvent 2 and once with 200 µl solvent 1. The peptides were eluted from the StageTips with 20 μl of elution buffer (5% NH_4_OH in 60% acetonitrile) and vacuum centrifuged until completely dry.

### Nano LC-MS and data analysis

Peptides were reconstituted in 10 µl of 0.05% trifluoroacetic acid (TFA), 4% acetonitrile and 5 µl were analyzed by an Ultimate 3000 reversed-phase capillary nano liquid chromatography system connected to a Q Exactive HF mass spectrometer (Thermo Fisher Scientific). Samples were injected and concentrated on a trap column (PepMap100 C18, 3 µm, 100 Å, 75 µm i.d.×2 cm, Thermo Fisher Scientific) equilibrated with 0.05% TFA in water. After switching the trap column inline, LC separations were performed on a capillary column (Acclaim PepMap100 C18, 2 µm, 100 Å, 75 µm i.d.×25 cm, Thermo Fisher Scientific) at an eluent flow rate of 300 nl/min. Mobile phase A contained 0.1% formic acid in water, and mobile phase B contained 0.1% formic acid in 80% acetonitrile and 20% water. The column was pre-equilibrated with 5% mobile phase B followed by an increase of 5–44% mobile phase B over 100 min. Mass spectra were acquired in a data-dependent mode utilizing a single MS survey scan (*m*/*z* 300–1650) with a resolution of 60,000, and MS/MS scans of the 15 most intense precursor ions with a resolution of 15,000. The dynamic exclusion time was set to 20 s and automatic gain control was set to 3×10^6^ and 1×10^5^ for MS and MS/MS scans, respectively. The mass spectrometry proteomics data were deposited to the ProteomeXchange Consortium via the PRIDE partner repository (Perez-Riverol et al., 2022) with the dataset identifier PXD051393.

MS and MS/MS raw data were analyzed using the MaxQuant software package (version 2.0.2.0) with implemented Andromeda peptide search engine (Tyanova et al., 2016a). Data were searched against the human reference proteome downloaded from Uniprot (79,684 proteins, taxonomy 9606, last modified April 18, 2022) using the default parameters except for enabling the options ‘label-free quantification (LFQ)’ and ‘match between runs’. Filtering and statistical analysis was carried out using the software Perseus version 1.6.14 (Tyanova et al., 2016b).

Only proteins which were identified with LFQ intensity values in all three replicates within at least one experimental group (for AP1µA–TurboID KI versus overexpression, Fig. 2) or in at least three out of four replicates (CLCa MS and AP complex interactome mapping, Figs 3, 5), were used for downstream analysis. Missing values were replaced from normal distribution (imputation) using the default settings in Perseus (width 0.3, down shift 1.8). For low abundance proteins (such as AP-4) sometimes many values need to be generated via imputation, which could lead to distortion of the datasets (Fig. S5). In our case, we believe that it was not an issue, but other methods to replace missing values could be used (Goeminne et al., 2020). Mean log_2_ fold protein LFQ intensity differences between experimental groups calculated in Perseus using unpaired two-tailed Student's *t*-tests with a permutation-based false discovery rate (FDR) of 0.05 to generate the adjusted *P*-values (q-values).

### Volcano plots

Volcano plots were created by plotting the −log_10_ *P*-value against the mean log_2_ fold protein LFQ intensity differences. The volcano plots in Fig. 2 show all proteins that were significantly enriched over the WT control. For the volcano plots in Figs 3 and 5, all proteins were included in the volcano plot representation to prevent exclusion of potential interactors for one of the two bait proteins that are compared.

### Identification of potential interactors of AP1µA comparing KI and overexpression

For identification of potential interactors only proteins that were significantly enriched (*P*<0.05 and log_2_ fold change >1) against both WT cells and cytosolic TurboID control were considered. These proteins were then screened with UniProt to identify proteins that were known to localize to either the Golgi or TGN, were thought to be involved in cellular trafficking, or were transmembrane proteins that might be trafficked by AP1µA or could control trafficking events (e.g. kinases and phosphatases). Together with all uncharacterized proteins these proteins were then considered potential interactors and were summarized in Tables S2 and S3.

### Identification of potential interactors and cargo proteins comparing different AP complexes

For identification of potential interactors and cargo proteins only proteins that were significantly enriched (*P*<0.05 and log_2_ fold change >1) compared to at least one other AP complex and also higher enriched than in the WT control (log_2_ fold change >0) were taken into consideration. These proteins were then screened with UniProt to identify proteins that were known to localize to either the Golgi and endosomal system (or to clathrin-coated pits in the case of AP-2), were thought to be involved in cellular trafficking, or were transmembrane proteins that were potential cargo proteins or could control trafficking events (e.g. kinases, phosphatases). All of those proteins that have at least one transmembrane domain were considered potential cargo proteins; all others were termed potential interactors. The results are summarized in Table S5 (switch between sheets for different AP complexes) and it was stated in which AP comparisons these proteins were identified.

### GO term analysis

For GO term analysis, only proteins were used that were significantly enriched (*P*-value <0.05 and log_2_ fold change>1) against both WT cells and cytosolic TurboID control (in case of KI versus OE, Fig. 2E) or against the WT control and the AP1µA/AP2µ sample (for V5-TurboID-CLCa, Fig. 5). GO Term analysis was performed using the GO::TermFinder open source software (Boyle et al., 2004).

## Supplementary Material



10.1242/joces.261952_sup1 Supplementary information

Table S1. List of quantified proteins detected by quantitaive mass spectrometry in proximity labeling-based experiments from HeLa cells expressing AP1μA fused to TurboID using endogenous tagging (knock-in, KI) or transient overexpression (OE). For control, a cytosolic TurboID overexpressed in wildtype cells (OE cytosolic TurboID) was used as well as biotin treated WT cells (WT). Each experiment was done in 3 replicates. Normalized log2 LFQ intensities according to the MaxQuant algorithm. Two-sample t-tests with permutation-based FDR control (FDR 0.05) were performed after imputation of missing values using the Perseus software platform.

Table S2.

Table S3.

Table S4. List of quantified proteins detected by quantitaive mass spectrometry in proximity labeling-based experiments from HeLa cells where the μ-subunit of the AP complexes AP-1 - AP-4 was tagged with TurboID. As control HeLa wildtype cells were used. Each experiment was done in 4 replicates. Normalized log2 LFQ intensities according to the MaxQuant algorithm. Two-sample t-tests (AP-1-AP-4 vs WT and AP vs AP) with permutation-based FDR control (FDR 0.05) were performed after imputation of missing values using the Perseus software platform.

Table S5.

Table S6. List of quantified proteins detected by quantitaive mass spectrometry in proximity labelling-based experiments from HeLa cells expressing CLCa-TurboID-V5 compared to AP1μA-TurboID-V5 and AP2μ-TurboID-V5. As negative control, biotin treated WT cells (WT) were used. Each experiment was done in 4 replicates. Normalized log2 LFQ intensities according to the MaxQuant algorithm. Two-sample t-tests with permutation-based FDR control (FDR 0.05) were performed after imputation of missing values using the Perseus software platform.
