# Supplementary material for: When less is more – a fast TurboID knock-in approach for high-sensitivity endogenous interactome mapping
Source: J Cell Sci. 2024 Aug 28;137(16):jcs261952. doi: 10.1242/jcs.261952 (PMC11385326; doi:10.1242/jcs.261952)
Supplement: Supplementary information [file joces-137-261952-s1.pdf]

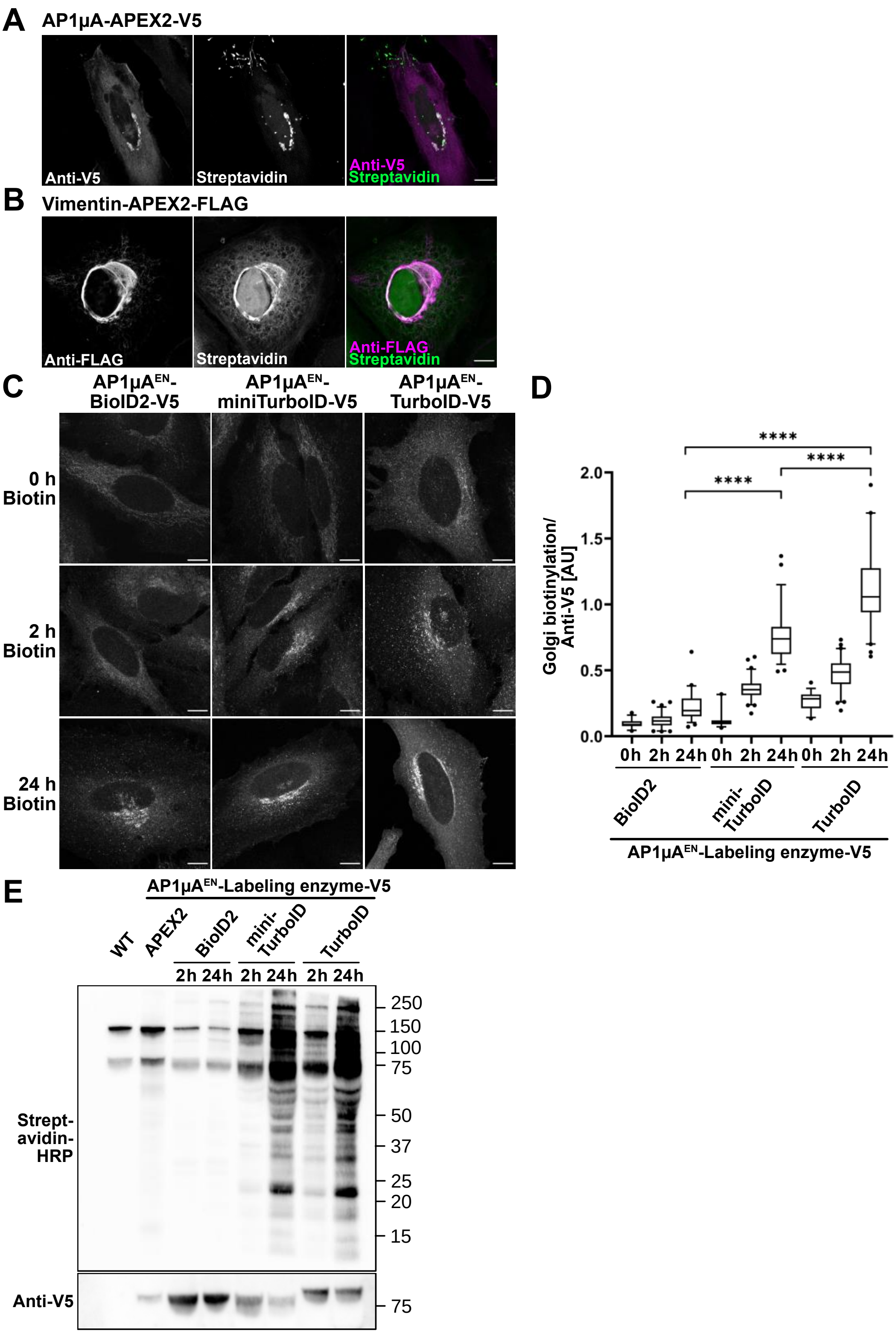

Fig. S1. Kinetics of labeling enzymes.

A: Cell transiently overexpressing AP1µA-APEX2-V5 were fixed and stained with an anti-V5 antibody to detect the fusion protein and streptavidin-AF488 to detect biotinylated proteins. Cells were incubated for 30 min with 500 µM biotin-phenol and labeling was induced for 1 min with H_2O_2. **B:** Cells transiently expressing

Journal of Cell Science • Supplementary information

Vimentin-APEX2-FLAG were fixed and stained with an anti-FLAG antibody to detect the fusion proteins and streptavidin-AF488 to detect biotinylated proteins. Cells were treated as described in **A**. **C:** Detection of biotinylated proteins with streptavidin-AF488 in fixed cells expressing different biotin ligases. 50 µM biotin was added for either 0 h, 2 h or 24 h before fixation. **D:** Ratio of biotinylated Golgi-localized proteins detected by streptavidin-AF488 to biotin ligase expression detected by anti-V5 antibody at the Golgi. Cells expressing the different biotin ligases endogenously fused to AP1µA were treated with 50 µM biotin for 0 h, 2 h or 24 h and then fixed and prepared for microscopy as in **C**. For each condition 30-50 cells were analyzed. All p-values from unpaired t-tests are <0.0001. Box plot whiskers 5-95 percentile. **E:** Comparison of the labeling efficiency of different labeling enzymes endogenously fused to AP1µA on a western blot. Cells were treated with 50 µM biotin for 2 h or 24 h, WT (wild type) cells were treated for 24 h with 50 µM biotin and APEX2 were incubated for 30 min with 500 µM biotin-phenol and labeling was induced for 1 min with H_2O_2. Whole cell lysates were blotted with streptavidin-HRP to detect biotinylated proteins, and anti-V5 antibody to compare ligase expression. This experiment was performed three times with similar results. **F:** The ratio of biotinylated proteins (Streptavidin-HRP) to the expressed labeling enzyme (Anti-V5) was calculated for each line on the blot in **E** and then normalized to the TurboID value for each of the three independent replicates.

Scale bars are 10 µm.

Journal of Cell Science • Supplementary information

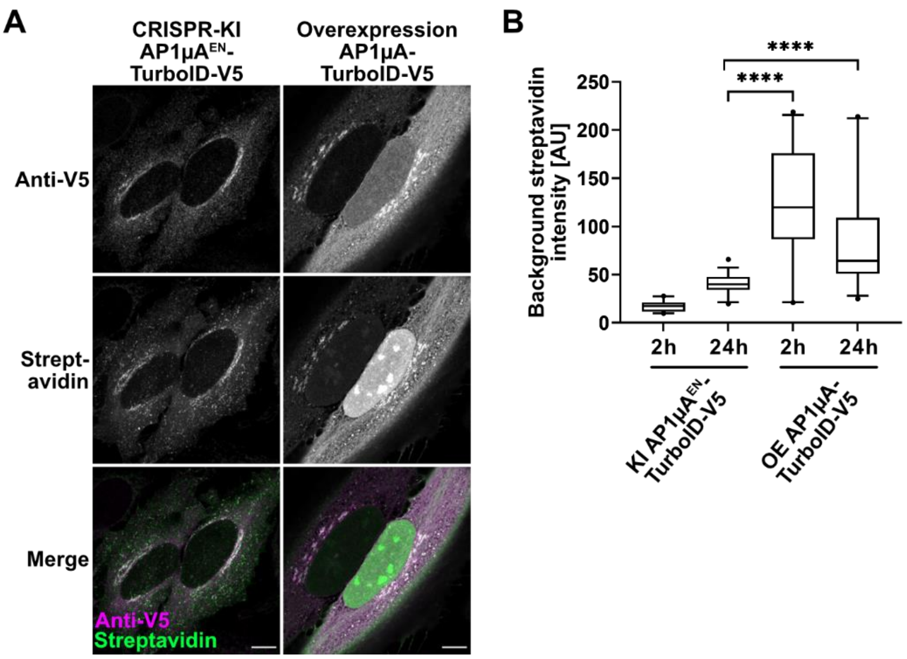

Fig. S2. Endogenous TurboID expression reduces unspecific labeling compared to transient overexpression.

A: Comparison of cells either endogenously expressing AP1μA^{EN}-TurboID-V5 or transiently overexpressing AP1μA-TurboID-V5. Cells were treated for 2 h with 50 μM biotin, fixed and stained with anti-V5 antibody to detect the labeling enzyme and streptavidin-AF488 to detect biotinylated proteins. **B:** Background biotinylation in cells either endogenously expressing AP1μA^{EN}-TurboID-V5 (KI) or transiently overexpressing AP1μA-TurboID-V5 (OE). Background biotinylation was measured in cells that were treated with biotin for 2 h or 24 h by measuring streptavidin-AF488 signal intensity from a cytosolic area. At least 20 cells were analysed per condition. P-values are all <0.0001. Scale bars are 10 μm.

Journal of Cell Science • Supplementary information

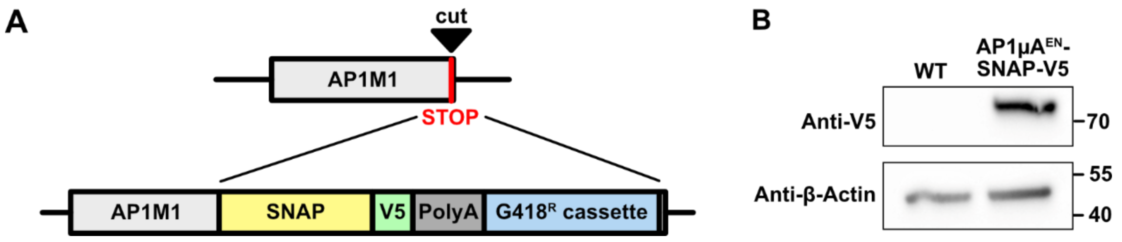

Fig. S3. Generation of an AP1µA^{EN}-SNAP-V5 cell line.

A: Scheme of KI strategy. AP1µA was C-terminally tagged with SNAP tag, a V5 tag and a resistance cassette that allows for rapid selection of positive cells. **B:** Blots of whole cell lysates to verify the AP1µA-SNAP-V5 KI by anti-V5 blotting.

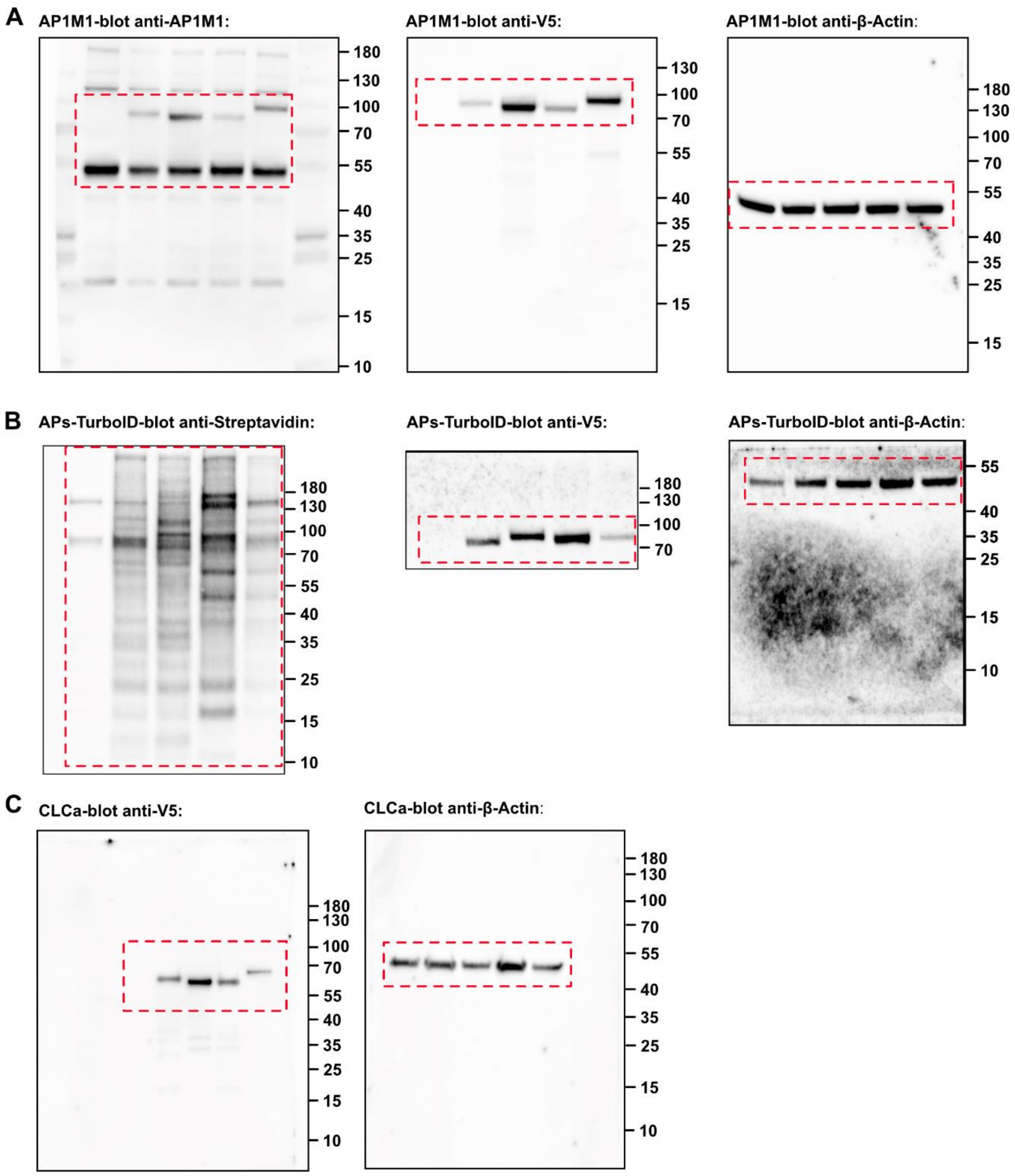

Fig. S4: Blot transparency.

A: Blot to show expression of different AP1µA-fusion proteins (from Fig. 1B). **B**: Blot to show fucntion and expression of different AP-TurboID-V5 fusions (from Fig. 3B). Anti-V5 and anti-β-actin blots were derived from the same blot membrane which was cut around 60 kDa. **C**: Blot to show expression of different CLCa-fusion proteins (from Fig. 4B).

Journal of Cell Science • Supplementary information

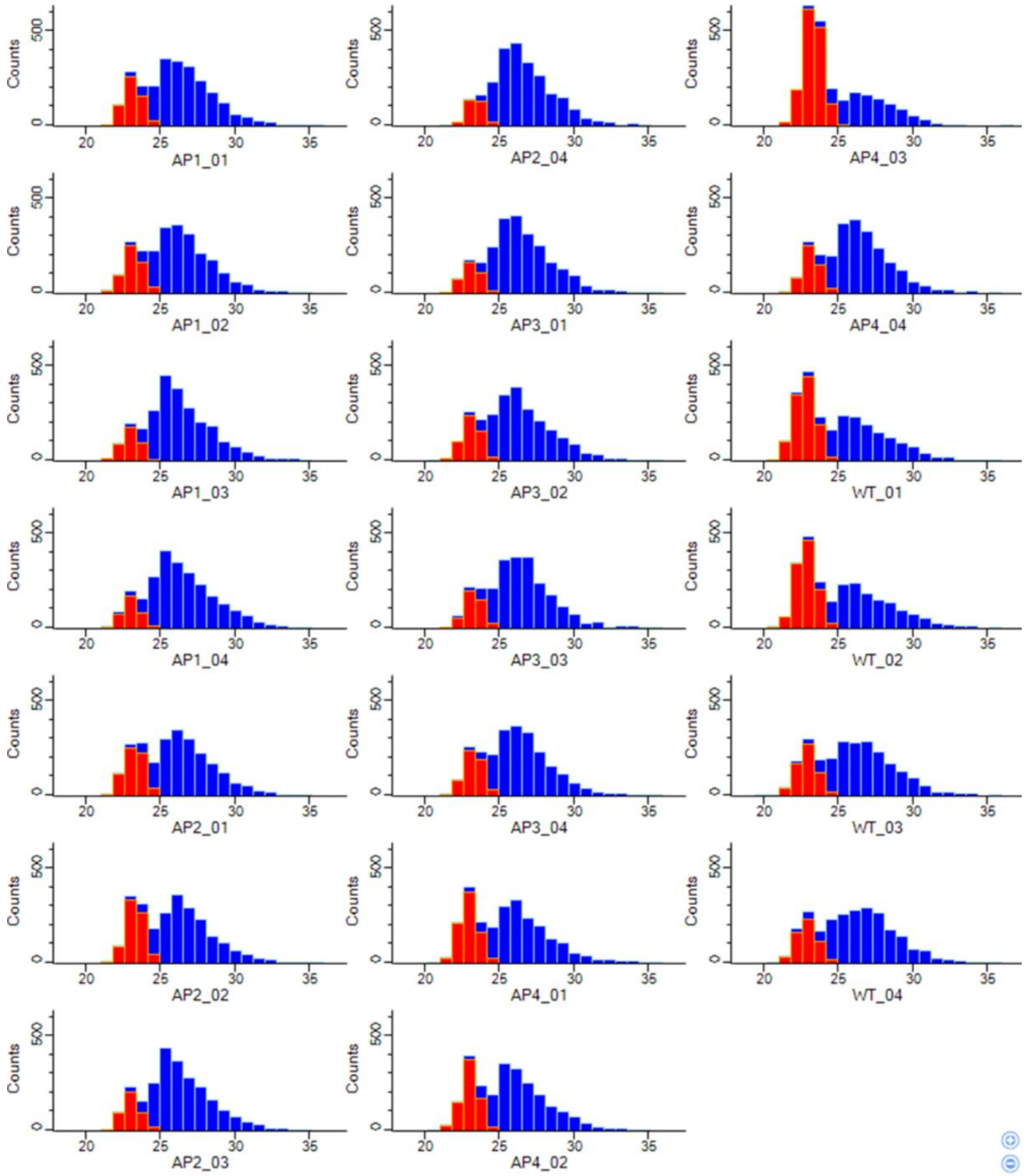

Fig. S5: Frequency distribution histograms after imputation.

Frequency distribution histograms of measured MS samples. Missing values that were replaced from normal distribution (imputation) using the default settings in Perseus (width 0.3, down shift 1.8) are shown in red. For high abundance proteins (AP-1, AP-2 and AP-3) distortion due to imputation was minimal.

Journal of Cell Science • Supplementary information

Table S1. List of quantified proteins detected by quantitaive mass spectrometry in proximity labeling-based experiments from HeLa cells expressing AP1μA fused to TurboID using endogenous tagging (knock-in, KI) or transient overexpression (OE). For control, a cytosolic TurboID overexpressed in wildtype cells (OE cytosolic TurboID) was used as well as biotin treated WT cells (WT). Each experiment was done in 3 replicates. Normalized log2 LFQ intensities according to the MaxQuant algorithm. Two-sample t-tests with permutation-based FDR control (FDR 0.05) were performed after imputation of missing values using the Perseus software platform.

Available for download at
https://journals.biologists.com/jcs/article-lookup/doi/10.1242/jcs.261952#supplementary-data

Table S2.

Available for download at
https://journals.biologists.com/jcs/article-lookup/doi/10.1242/jcs.261952#supplementary-data

Table S3.

Available for download at
https://journals.biologists.com/jcs/article-lookup/doi/10.1242/jcs.261952#supplementary-data

Table S4. List of quantified proteins detected by quantitaive mass spectrometry in proximity labeling-based experiments from HeLa cells where the μ-subunit of the AP complexes AP-1 - AP-4 was tagged with TurboID. As control HeLa wildtype cells were used. Each experiment was done in 4 replicates. Normalized log2 LFQ intensities according to the MaxQuant algorithm. Two-sample t-tests (AP-1-AP-4 vs WT and AP vs AP) with permutation-based FDR control (FDR 0.05) were performed after imputation of missing values using the Perseus software platform.

Available for download at
https://journals.biologists.com/jcs/article-lookup/doi/10.1242/jcs.261952#supplementary-data

Table S5.

Available for download at
https://journals.biologists.com/jcs/article-lookup/doi/10.1242/jcs.261952#supplementary-data

Table S6. List of quantified proteins detected by quantitaive mass spectrometry in proximity labelling-based experiments from HeLa cells expressing CLCa-TurboID-V5 compared to AP1µA-TurboID-V5 and AP2µ-TurboID-V5. As negative control, biotin treated WT cells (WT) were used. Each experiment was done in 4 replicates. Normalized log2 LFQ intensities according to the MaxQuant algorithm. Two-sample t-tests with permutation-based FDR control (FDR 0.05) were performed after imputation of missing values using the Perseus software platform.

Available for download at
https://journals.biologists.com/jcs/article-lookup/doi/10.1242/jcs.261952#supplementary-data

Journal of Cell Science • Supplementary information

Table S7. Antibody and Streptavidin conjugates.

**Antibody/Conjugate**	**Supplier**	**Catalogue Number**	**Used concentration**
V5-Tag (D3H8Q) Rabbit	Cell Signaling	13202	1:1000 for IF and WB
DYKDDDDK Tag (D6W5B) Rabbit	Cell Signaling	14793	1:1000 for IF
β-Actin (8H10D10) Mouse	Cell Signaling	3700	1:1000 for WB
Anti-AP1M1 Rabbit	Abcam	ab230273	1:1000 for WB
Anti-CHC Mouse	Novus Biologicals	102130-888	1:1000 for IF
Anti-p230 Mouse	BD transduction laboratories	AB_398809	1:1000 for IF
Anti-Rabbit-Atto647N Goat	Sigma-Aldrich	40839	1:1000 for IF
Anti-Mouse-Alexa594 Goat	Invitrogen	A66790	1:1000 for IF
Anti-Rabbit- HRP Goat	Abcam	ab6721	1:5000 for WB
Anti-Mouse- HRP Goat	Abcam	ab6789	1:5000 for WB
Streptavidin, HRP conjugate	Thermo Scientific	N100	1:4000 for WB
Streptavidin, Alexa Fluor™ 488 conjugate	Invitrogen	S11223	1:1000 for IF
Streptavidin, STARORANGE conjugate	Abberior	STORANGE-0120	1:500 for IF

Table S8. Primer sequences.

Primer Name	Primer Sequence (5'-3')
**PolyA sequence:**	
PolyA NheI sense	AGTTCGCTAGCCCGCGACTCTAGATCATAATCAGC
PolyA NotI antisense	TGCACGCGGCCGCCTTACAATTTACGCCTTAAGATACA
**G418 cassette for C-terminal KI:**	
G418 NotI sense	CGTCGGCGGCCGCCCTGAGGCGGAAAGAACCAGCTGTGGAAT GTGTGTCAGTTAG
G418 EcoRI antisense	TCATGGAATTCTTTATTCTGTCTTTTTATTGCCGTC
**Labeling enzymes for C-terminal KI:**	
APEX2 BamHI sense	GATGGGGATCCATGGGAAATCATACCCAACAGTGTCCG
APEX2 V5 NheI antisense	CTAACGCTAGCTCAGGTGCTGTCCAGGCCCAGCAGGGGGTTG GGGATGGGCTTGCCGGCGTCGGCAAATCCCAGTTCTGAC
BioID2 BamHI sense	GATGGGGATCCATGTTCAAGAACCTGATCTGGCTGAAGG
BioID2 V5 NheI antisense	CTAACGCTAGCTCAGGTGCTGTCCAGGCCCAGCAGGGGGTTG GGGATGGGCTTGCCGCTTCTTCTCAGGCTGAACTCGCCG
TurboID BamHI sense	GATGGGGATCCATGAAAGACAATACTGTGCCTCTGAAGC
MiniTurboID BamHI sense	GATGGGGATCCATGATCCCGCTGCTGAACGCTAAACAGA
TurboID/MiniTurboID V5 NheI antisense	CTAACGCTAGCTCAGGTGCTGTCCAGGCCCAGCAGGGGGTTG GGGATGGGCTTGCCCTTTTCGGCAGACCGCAGACTGATT
**SNAP primer for C-terminal KI:**	
SNAP BamHI sense	CTGATGGATCCGACAAAGACTGCGAAATGAAGCGCA
SNAP V5 NheI antisense	TACAAGCTAGCTCAGGTGCTGTCCAGGCCCAGCAGGGGGTTG GGGATGGGCTTGCCACCCAGCCCAGGCTTGCCCAGTCTG
**G418 cassette with loxP sites for N-terminal KI:**	
G418 loxP NheI sense	ATGTCGCTAGCATAACTTCGTATAGCATACATTATACGAAGTTAT CCTGAGGCGGAAAGAACCAGCTGTGGAATGTGTGTCAGTTAG
G418 loxP BamHI antisense	TGCACGGATCCGTGATAACTTCGTATAATGTATGCTATACGAAG TTATTTTATTCTGTCTTTTTATTGCCGTCATAGCGCGGGTT
**Labeling enzymes for N-terminal KI:**	
APEX2 EcoRI sense	CTAACGAATTCGGGAAATCATACCCAACAGTGTCCG
APEX2 SpeI antisense	CTAACACTAGTGGCGTCGGCAAATCCCAGTTCTGAC
BioID2 EcoRI sense	CTAACGAATTCTTCAAGAACCTGATCTGGCTGAAGG
BioID2 SpeI antisense	CTAACACTAGTGCTTCTTCTCAGGCTGAACTCGCCG
TurboID EcoRI sense	CTAACGAATTCAAAGACAATACTGTGCCTCTGAAGC
MiniTurboID EcoRI sense	CTAACGAATTCATCCCGCTGCTGAACGCTAAACAGA
TurboID/ MiniTurboID SpeI	GTCACACTAGTCTTTTCGGCAGACCGCAGACTGATT

Journal of Cell Science • Supplementary information

antisense	
**Overexpression plasmids:**	
APEX2 BamHI sense	CTAACGGATCCGGGAAATCATACCCAACAGTGTCCG
APEX2 V5 NotI antisense	CTAACGCGGCCGCTCAGGTGCTGTCCAGGCCCAGCAGGGG
TurboID BamHI sense	AGTCAGGATCCATGAAAGACAATACTGTGCCTCTGAAGCTG
TurboID V5 NotI antisense	TAGCTGCGGCCGCTCAGGTGCTGTCCAGGCCCAGCAGGGGGT TGGGGATGGGCTTG
AP1μA EcoRI sense	GATGGGAATTCATGTCCGCCAGCGCCGTCT
AP1μA linker BamHI antisense	TGTGCGGATCCTGAGCCGGAACCAGAGCCTGACCCT
SCYL2 EcoRI sense	TACCGGAATTCTATGGAGTCCATGCTTAATAA
SCYL2 BamHI antisense	TACCGGGATCCTCACCCAAAAAGATCTTTTAAAT
