# Supplementary material for: When less is more – a fast TurboID knock-in approach for high-sensitivity endogenous interactome mapping
Source: J Cell Sci. 2024 Aug 28;137(16):jcs261952. doi: 10.1242/jcs.261952 (PMC11385326; doi:10.1242/jcs.261952)
Supplement: Supplementary file 1 [file joces-137-261952_review_history.pdf]

When less is more - a fast TurboID knock-in approach for high-sensitivity endogenous interactome mapping

Alexander Stockhammer, Carissa Spalt, Antonia Klemt, Laila S. Benz, Shelly Harel, Vini Natalia, Lukas Wiench, Christian Freund, Benno Kuropka and Francesca Bottanelli

DOI: 10.1242/jcs.261952

Editor: Jennifer Lippincott-Schwartz

Review timeline

Original submission:	11 January 2024
Editorial decision:	20 February 2024
First revision received:	5 May 2024
Editorial decision:	20 June 2024
Second revision received:	24 June 2024
Accepted:	10 July 2024

Original submission

First decision letter

MS ID#: JOCES/2024/261952

MS TITLE: When less is more - A fast TurboID KI approach for high sensitivity endogenous interactome mapping

AUTHORS: Alexander Stockhammer, Carissa Spalt, Laila Benz, Shelly Harel, Vini Natalia, Lukas Wiench, Christian Freund, Benno Kuropka, and Francesca Bottanelli
ARTICLE TYPE: Tools and Resources

We have now reached a decision on the above manuscript.

To see the reviewers' reports and a copy of this decision letter, please go to: https://submit-jcs.biologists.organd click on the 'Manuscripts with Decisions' queue in the Author Area. (Corresponding author only has access to reviews.)

As you will see, the reviewers gave favourable reports but raised some critical points that will require amendments to your manuscript. Please respond directly to all of the concerns raised by reviewer #1. Regarding reviewers 2 and 3, please only address the comments you feel will substantially improve the take-home message of your paper. Many of their comments require a lot of additional work and won't substantially improve your work, so please only do what you think is necessary.

Please ensure that you clearly highlight all changes made in the revised manuscript. Please avoid using 'Tracked changes' in Word files as these are lost in PDF conversion.

I should be grateful if you would also provide a point-by-point response detailing how you have dealt with the points raised by the reviewers in the 'Response to Reviewers' box. Please attend to all of the reviewers' comments. If you do not agree with any of their criticisms or suggestions please explain clearly why this is so.

Reviewer 1

© 2024. Published by The Company of Biologists under the terms of the Creative Commons Attribution License (https://creativecommons.org/licenses/by/4.0/).

Advance summary and potential significance to field

This manuscript presents a side-by-side comparison of different proximity labeling enzymes and constructs (knock-in versus overexpression) for the clathrin adaptor protein complex-1 (AP1). It shows that a knockin of turboID produces more labeling than other enzymes and that the knockin of turboID shows more selective labeling than overexpression. Similar, findings are presented for a knockin of N-terminal tag of clathrin light chain. Proteomic datasets are collected for AP1, AP2, AP3, AP4 and clathrin light chain allowing the identification of adaptor-specific interactors. Some of these are validated for AP1. Overall, the paper presents a broadly important advance in proximity labeling by demonstrating the effectiveness of different enzyme and expression strategies. The proteomics datasets are exciting for researchers in the field of membrane traffic. I think the work is significant and the quality of the work is high.

Comments for the author

Overall, I have only minor comments.

In the first paragraph of the results, I was not entirely certain how positively edited cells were identified without reading the materials and methods. It would be useful to say that the efficiency was determined as the number of V5+ cells in the results. Also, the estimation of heterozygous cells is estimated from a western blot. I am a little unclear on what analysis was done here. My guess is that based on the % efficiency the and the intensity of the tagged versus endogenous was used. A brief description would be useful. It may also be possible to look at V5 intensity of the cells to show that few had 2x intensity.

On line 315, the phrasing "cause it not be transcribed." was a bit awkward I might use the phrase 'prevent its transcription'

Reviewer 2

Advance summary and potential significance to field

In this manuscript, the authors present their strategy to quickly select a pool of knock-in HeLa cell lines that express proteins genetically tagged at their endogenous locus with proximity-dependent biotinylation (PDB) enzymes used for proteomics studies. With the example of AP1&[mu]A protein, involved in intracellular protein trafficking, they show that proximity-proteomics datasets obtained from endogenous tagging are more faithful than corresponding datasets from the same tagged protein but expressed after the transient transfection of a plasmid, leading to overexpression. The manuscript is written in a clear and logical manner.

There are already numerous strategies to select genome-engineered cells, including pools of engineered cells (e.g. Singh, S. et al., Sci Rep 13, 1719 (2023) or Stoyko D, et al., Curr Protoc. 2(12)(2022), and there are many more). It is also the general recommendation when performing PDB-proteomics that the fusion protein between the protein of interest and the labeling enzyme is tested to check if it behaves (expression, localization, function) like its endogenous counterpart (see e.g. Qin, W., et al. Nat Methods 18, (2021) or Sears RM, et al., Methods Mol Biol. 2012, (2019)). So as mentioned by the authors, scientists who perform PDB-proteomics do already follow different strategies to control expression levels of their fusion proteins. These strategies, as the one presented here, have advantages and drawbacks.

Hence the concepts behind the current manuscript are not per se novel but they are nicely combined to showcase their application to proteins involved in intracellular protein trafficking. In particular, the use of the datasets to find proteins associated and cargo proteins of different AP complexes mediated transport route is convincingly shown. In my opinion, the main interest of the manuscript is thus less the technical part than the datasets themselves.

Comments for the author

© 2024. Published by The Company of Biologists under the terms of the Creative Commons Attribution License (https://creativecommons.org/licenses/by/4.0/).

For publication of this manuscript I recommend the following revisions. I apologize for the long list but I truly believe all these points can be addressed in a timely manner with a reasonable amount of resources:

ESSENTIAL REVISIONS

1. The point about the different stability of the various fusion proteins is important: lower levels of expression may be due to selection against cells with elevated expression and/or rapid degradation of the corresponding labeling enzymes. These toxicity and/or instability issues may lead to experiments performed in unhealthy cells which can confound the resulting datasets. At the moment the data do not allow drawing conclusions, but this can be easily addressed by isolating a couple of single clones from the pools of cells expressing the APEX2, BioID2, miniTurbo and TurboID fusions. How many alleles were successfully targeted in these clones can be easily analyzed and clones with equal number of KI-alleles can be analyzed for expression of the fusion proteins by western blot. With this, how stable the different fusion proteins are can be directly assessed. This is important not only for the quality of the proteomics datasets but also to reliably assess KI rates from microscopy data (see points 12/13).

2. Sup Fig. 1, please add data for the 0h time point (missing here), there appears to be quite some non-Golgi staining for biotin for TurboID at 2h and 24h but it is hard to judge without a comparison for 0h biotinylation and WT cells.

3. Fig. 4B: please include a blot for CLCa, this would also help appreciate why the amount of V5-TurboID seems to be enormously higher than that of the other enzymes (which is not commented by the authors).

4. Fig. 3A: a negative control (WT cells treated with the same antibody/streptavidin, and image processed with same parameters) is needed

5. lines 403/406: if the datasets obtained in this study are meant to serve as “starting point for studies aimed at unravelling the mechanisms of (…) AP complexes” then the MS data should be analyzed properly:

For the bioinformatic analysis of the MS data imputations were used with the Perseus package to replace missing values. As the number missing values in typical proximity-dependent biotinylation experiments is typically quite high and do not only represent missing completely at random and missing at random values (also because sample and negative control are quite different samples), imputation of missing values is typically not valid for such datasets as it strongly distorts the observed frequency distributions. If the authors believe this was a valid approach for their specific datasets, they should provide frequency distribution histograms of log2-transformed protein levels before and after imputations to prove their point. Otherwise I can recommend referring to Goeminne L et al., Anal. Chem. 2020, 92, 6278−6287 (see Fig. 1 for how imputation can strongly distort the dataset) and also Wolski W et al., J. Proteome Res. 2023, 22, 1092−1104 for alternative more suitable strategies to address missing values. The clearest hits will still be identified but the rest of the datasets will most probably be affected which will be important for future studies that may use the present datasets as a starting point.

6. For all blots: please provide in the supplementary material images of the uncropped blots used to produce the figures and indicate the boxes that were used to quantify the signal for V5 and biotinylation.

7. For all blots: a loading control is missing and makes the quantifications unreliable, especially since the linearity of the quantified signals is not demonstrated.

ADDITIONAL COMMENTS:

8. In the same line to point 7, since the quantification of the blots is based on ECL signals measured in triplicates each time from two separate blots (one for biotinylation, one for V5), the resulting data are not sufficient at all to make serious statistics (small amount of data points, technical methods prone to huge variations, ECL signals with limited linear range). Hence on Sup. Fig. 1F I recommend showing the bar for the average value and the corresponding three data points

© 2024. Published by The Company of Biologists under the terms of the Creative Commons Attribution License (https://creativecommons.org/licenses/by/4.0/).

for each enzyme but no error bar and no mention of (non) significance. One can see by eye how the data look like and the statistics are of very limited relevance here.

9. In the first paragraph of the result section, please clearly state what cell type was used in this study (in the present version it only appears in some of the figure legends and in the supplementary material).

10. The description of the strategy to obtain a pool of knock-in cells is not detailed enough. Please indicate on your schemes (Fig. 1A and Fig. 4A) what you refer to as the “resistance cassette” is made of (Promoter / polyA signal or IRES / polyA signal of native locus?). For the sake of reproducibility I would also recommend providing in the supplemental material .gb files for the KI constructs as it is much easier than trying to reconstruct a similar plasmid following the instructions given in the supplementary material.

11. Lines 101-109. The timeline of the experiments is not clear: G418 is applied 3 days after transfection until the cells are confluent and then the cells are analyzed. How many days is then G418 applied to the cells, how was it checked that this time window is long enough to kill all the cells that did not undergo HDR? If there is no data supporting this the estimated KI rates are not really telling.

12. For the quantification of the KI rates, the method description is too succinct to be reproduced by others (what parameters/threshold were used in the microscopy experiments). Rather than using microscopy I recommend using flow cytometry to measure KI rates, FC is much more powerful in term of unbiased quantification and will give the important information about the homogeneity of the expression of the fusion protein within the selected the cell pool.

13. By reading the manuscript I understand that the KI rate is defined as the % of cells in which a V5 signal could be detected by microscopy after few days of G418 selection. The western blot data do not really correlate with the microscopy data (especially for the CLC KI cells), which may be due to different stability of the various fusion proteins. This point which is independent of the KI rate per se is only commented briefly in the discussion (line 352) but with a confusing wording: the KI rate (i.e. rate of foreign DNA integration in the host genome) can not be influenced by “lower stability of (…) fusion proteins”, these are two different things.

14. Lines 110/111: strictly speaking since you have heterozygous cells the enzyme are not expressed at the endogenous levels of the target proteins but below endogenous levels.

15. Line 132/134: “The low biotinylation rate of BioID2 makes it, in our opinion, unfavorable compared to the TurboID variants, as a high amount of biotinylation is crucial to enrich enough protein for the subsequent identification and quantification by mass spectrometry” the statement is rightfully presented as an opinion but can still be understood as a recommendation. I would recommend using a wording that makes it clear that this opinion is not a general recommendation as in other contexts the higher amount of biotinylation proved to lead to less specific datasets (see May D, Cells 2020 in which this was experimentally tested). In addition, the biotinylation signals shown here are before performing streptavidin pulldowns so the amount of biotinylation obtained with BioID2 would have been definitely enough for the subsequent MS analysis.

16. Lines 195/196: Fig. 2E shows p-values of GO term enrichment, not fold changes, so the wording “more enriched” appears to be incorrect to describe the differences observed.

17. In the comparison OE vs KI (Fig. 2G) quite a lot of hits are also enriched in the OE conditions. Do these proteins (possibly after applying a filtering with the cyt TurboID dataset) correspond to anything of significance for AP-1 biology (maybe cytosolic partners of the complex when it is not associated with the TGN)?

18. Fig. 3A: any explanation why there is such a strong accumulation of biotinylated proteins in the nucleus of AP3A-muA-turboID expressing cells?

19. Fig. 3E: the whole point of the manuscript is that one should not work with overexpressed proteins but for the validation presented here, the potential interactors are overexpressed. Since

© 2024. Published by The Company of Biologists under the terms of the Creative Commons Attribution License (https://creativecommons.org/licenses/by/4.0/).

overexpression of cargo proteins has been described to potentially affect their transport route (see the example of SERINC3, a cargo of AP-4 in Davies A et al., 2018), is there any way to use antibodies against endogenous ITGB1, VAMP7 and SCYL2 instead of transient transfection of the GFP-tagged proteins?

20. Lines 211/213: “Taken together, our findings highlight that tagging the protein with the labelling enzyme endogenously increases the sensitivity of the proximity biotinylation and the MS measurement so that very transient but specific interactors, such as specific cargo proteins, can be identified.” The wording is misleading: it is actually the expression level (less than physiological vs overexpressed) that matters not how it is achieved (KI). As the authors very well describe in their introduction, overexpressing the target protein is not the recommended approach as this leads to artefacts such as mislocalization. Hence most PDB studies are not performed after transient transfection but use various strategies to achieve physiological levels. The KI approach is one possibility but not the only one.

21. For the part on the AP-4 dataset interactors (lines 264-266), it would be good to compare the dataset obtained here with the dataset from Ref. 49, which used a completely different approach (the discussion would be a good place for this). In Supplementary Table 4, it appears that the comparison AP-4 vs. WT cells led to the identification of no significant hits for AP-4, this is not commented in the main text.

22. Line 368/369: “to prevent background biotinylation of TurboID, cells can be grown in biotin-free media 72h prior to the experiment”. I would be very careful giving this advice without supporting data as a general fix for the background of TurboID as it may not be suited to all test systems (see e.g. Collins J. et al., JBC 1988 or Elahi A et al., J Immunol 2018). Labeling with heavy biotin and detection of heavy biotin-modified peptides is probably a safer approach to tackle this problem.

23. The limitations of the KI approach are not at all discussed: obviously 3’UTR are strongly modified (shortened) with the potential of losing important post-transcriptional regulation elements (which may be important for gene in e.g. cell differentiation test sytems). Any other potential limitation should be mentioned.

24. The limitations of the PDB approach applied to the identification of cargo proteins for AP-mediated transport routes are also not addressed (e.g.: soluble cargo transported inside tubular vesicular structure are not accessible for biotinylation; transmembrane cargo proteins with short cytoplasmic tails may not have lysine residues as potential acceptors for biotinylation). A balanced discussion and mention of alternative/complementary approaches is needed.

25. The MS data should be uploaded to a public repository

Reviewer 3

Advance summary and potential significance to field

In their manuscript, Stockhammer et al. present a rapid approach for fusion of biotinylating enzymes at the N- and C-termini of proteins via knock-in at their endogenous genetic locus, enabling faster and more sensitive interactome mapping of any protein. Their method utilizes CRISPR-Cas9 homology-directed repair combined with an antibiotic selection cassette, eliminating the need for clonal propagation and analysis during cell engineering and selection. The authors demonstrate the effectiveness of their approach by tagging the C-termini of AP complexes 1-4, revealing both known interactors and cargo proteins, along with potential novel ones. They also introduce an endogenous editing strategy for N-terminal knock-ins, validated through mapping the interactome of the clathrin light chain. By comparing the interactomes of different AP complexes and clathrin, the authors identify specific interactors and biological cargoes associated with each protein complex.

Comments for the author

© 2024. Published by The Company of Biologists under the terms of the Creative Commons Attribution License (https://creativecommons.org/licenses/by/4.0/).

A protein's interactome is a key determinant of its biologic role. A common approach to mapping protein interactomes is proximity ligation, in which fusion to a labeling enzyme enables tagging of proximal interacting proteins, which are then detected and quantified by mass spectrometry. Proximity ligation has historically faced challenges such as high assay noise from over-expression of labeling enzymes or complex genetic engineering requirements. A simplified method for generating endogenous genetic fusions with labeling enzymes would greatly aid in interactome mapping and functional understanding of proteins.

In their manuscript, Stockhammer et al. present a rapid approach for fusion of biotinylating enzymes at the N- and C-termini of proteins via knock-in at their endogenous genetic locus, enabling faster and more sensitive interactome mapping of any protein. Their method utilizes CRISPR-Cas9 homology-directed repair combined with an antibiotic selection cassette, eliminating the need for clonal propagation and analysis during cell engineering and selection. The authors demonstrate the effectiveness of their approach by tagging the C-termini of AP complexes 1-4, revealing both known interactors and cargo proteins, along with potential novel ones. They also introduce an endogenous editing strategy for N-terminal knock-ins, validated through mapping the interactome of the clathrin light chain. By comparing the interactomes of different AP complexes and clathrin, the authors identify specific interactors and biological cargoes associated with each protein complex.

Comments and Concerns:

1. The presented approach may lack significant novelty. The supposed advancement of the approach is the inclusion of an antibiotic selection cassette, a technique commonly employed for selecting genetic knock-in cells.

2. Knock-in efficiency for each biotinylating enzyme is mentioned in the text but it is unclear exactly what these percentages represent. I.e., do state percentages indicate the percentage of alleles with integrations or the percentage of cells with at least one integration? Were these calculated pre- or post- selection with the antibiotic?

3. The authors do not provide validation that TurboID fusion to APμ subunits has no effect on AP complex formation and function. The authors should provide a supplementary figure demonstrating that AP complex function continues at normal levels.

4. The STED data in figure 1 appears to demonstrate that there are many nanodomains for which biotinylation and expression for the enzyme do not overlap. Do the authors have an explanation for this observation?

5. The authors chose 24 hour biotin labeling for interactome mapping. 24 hour labeling generates significantly more labeled proteins than 2 hour labeling based on western blot analysis (supplementary figure 1) and I am concerned that this increase in signal may reflect primarily higher levels of off-target labeling rather than on-target labeling. In a supplementary figure, the authors should include an analysis demonstrating that background/cytosolic biotin labeling is still minimal when TurboID labeling proceeds for 24 hours. Additionally, I wonder how MS results differ between 2 and 24 hours – are major known interactors missing?

6. The authors validate the possible novel interactors of AP-1 (ITGB1, VAMP7, and SCYL2) using co-localization by fluorescence microscopy. While there are points of co-localization between AP-1 and these proteins, there is no control condition, making it difficult to determine if co-localization events are biologically significant or simply chance overlap. The authors should perform a control with a non-interactor of AP1, and quantify and compare the colocalization of AP1 to each novel interactor and the control. Alternatively, the authors could perform a co-IP as validation.

7. In Figure 3, the authors identify interactors and/or cargo proteins of the AP1 complexes by comparing protein abundances between the AP complexes. For complexes that do not localize to the same subcellular space (e.g. AP1 and AP2), the landscape of non-specific labeling for these proteins may differ significantly causing off-target proteins to become significantly enriched. Instead of comparing across AP complexes, the authors should have compared to a control that

© 2024. Published by The Company of Biologists under the terms of the Creative Commons Attribution License (https://creativecommons.org/licenses/by/4.0/).

localizes to the same subcellular compartment and is likely to have a similar landscape of non-specific labeling.

8. Why do the authors validate the N-terminal fusion on clathrin as opposed to AP-1? I would be interested to see if similar results are achieved for N- and C-terminal fusions of AP-1.

9. The N-terminal TurboID fusion appears to generate higher background biotinylation (figure 4)? How do the authors explain this and are they concerned by this potentially higher background signal?

First revision

Author response to reviewers' comments

We would like to thank all the reviewers for their feedback on the article. We think by addressing their concerns we were able to improve on the latest version.

Reviewer 1 Advance Summary and Potential Significance to Field:
This manuscript presents a side-by-side comparison of different proximity labeling enzymes and constructs (knock-in versus overexpression) for the clathrin adaptor protein complex-1 (AP1). It shows that a knockin of turboID produces more labeling than other enzymes and that the knockin of turboID shows more selective labeling than overexpression. Similar, findings are presented for a knockin of N-terminal tag of clathrin light chain. Proteomic datasets are collected for AP1, AP2, AP3, AP4 and clathrin light chain allowing the identification of adaptor-specific interactors. Some of these are validated for AP1. Overall, the paper presents a broadly important advance in proximity labeling by demonstrating the effectiveness of different enzyme and expression strategies. The proteomics datasets are exciting for researchers in the field of membrane traffic. I think the work is significant and the quality of the work is high.

Reviewer 1 Comments for the Author:
Overall, I have only minor comments.

In the first paragraph of the results, I was not entirely certain how positively edited cells were identified without reading the materials and methods. It would be useful to say that the efficiency was determined as the number of V5+ cells in the results. Also, the estimation of heterozygous cells is estimated from a western blot. I am a little unclear on what analysis was done here. My guess is that based on the % efficiency the and the intensity of the tagged versus endogenous was used. A brief description would be useful. It may also be possible to look at V5 intensity of the cells to show that few had 2x intensity.

We thank the reviewer for the suggestion. We changed the manuscript, to be make more clear how the estimated KI efficiencies were calculated.

Line 112:
"Homology-directed repair is a rare event, mainly yielding a mixed population of heterozygous cells, therefore we assume most of the generated cells are heterozygous. To estimate the efficiency of each KI, we checked for V5 expression using immunofluorescence microscopy to detect positively edited cells and found that 43% (APEX2), 63% (BioID2), 45% (MiniTurboID), 59% (TurboID) of the cells expressed the fusion protein."

We removed the sentence regarding the western blot from the manuscript to avoid misunderstandings.

© 2024. Published by The Company of Biologists under the terms of the Creative Commons Attribution License (https://creativecommons.org/licenses/by/4.0/).

Removed sentence: “After comparison to the expression levels of edited AP1μA on the western blot, we assume that most of the cells in our population are heterozygous for AP1μA fusion protein expression.”

When we quantified the amount of V5 signal in cells (Fig. S1D), we found that the V5 intensities of V5 signals were all in a similar range, suggesting that the vast majority of the cells is, as expected, heterozygous.

On line 315, the phrasing "cause it not be transcribed." was a bit awkward I might use the phrase 'prevent its transcription'

We changed the text as suggested.

*****

Reviewer 2 Advance Summary and Potential Significance to Field:

In this manuscript, the authors present their strategy to quickly select a pool of knock-in HeLa cell lines that express proteins genetically tagged at their endogenous locus with proximity-dependent biotinylation (PDB) enzymes used for proteomics studies. With the example of AP1&[mu]A protein, involved in intracellular protein trafficking, they show that proximity-proteomics datasets obtained from endogenous tagging are more faithful than corresponding datasets from the same tagged protein but expressed after the transient transfection of a plasmid, leading to overexpression. The manuscript is written in a clear and logical manner.

There are already numerous strategies to select genome-engineered cells, including pools of engineered cells (e.g. Singh, S. et al., Sci Rep 13, 1719 (2023) or Stoyko D, et al., Curr Protoc. 2(12)(2022), and there are many more). It is also the general recommendation when performing PDB-proteomics that the fusion protein between the protein of interest and the labeling enzyme is tested to check if it behaves (expression, localization, function) like its endogenous counterpart (see e.g. Qin, W., et al. Nat Methods 18, (2021) or Sears RM, et al., Methods Mol Biol. 2012, (2019)). So as mentioned by the authors, scientists who perform PDB-proteomics do already follow different strategies to control expression levels of their fusion proteins. These strategies, as the one presented here, have advantages and drawbacks.

Hence the concepts behind the current manuscript are not per se novel but they are nicely combined to showcase their application to proteins involved in intracellular protein trafficking. In particular, the use of the datasets to find proteins associated and cargo proteins of different AP complexes mediated transport route is convincingly shown. In my opinion, the main interest of the manuscript is thus less the technical part than the datasets themselves.

Reviewer 2 Comments for the Author:

For publication of this manuscript I recommend the following revisions. I apologize for the long list but I truly believe all these points can be addressed in a timely manner with a reasonable amount of resources:

ESSENTIAL REVISIONS

1. The point about the different stability of the various fusion proteins is important: lower levels of expression may be due to selection against cells with elevated expression and/or rapid degradation of the corresponding labeling enzymes. These toxicity and/or instability issues may lead to experiments performed in unhealthy cells which can confound the resulting datasets. At the moment the data do not allow drawing conclusions, but this can be easily addressed by isolating a couple of single clones from the pools of cells expressing the APEX2, BioID2, miniTurbo and TurboID fusions. How many alleles were successfully targeted in these clones can be easily analyzed and clones with equal number of KI-alleles can be analyzed for expression of the fusion proteins by western blot. With this, how stable the different fusion proteins are can be directly assessed. This is important not only for the quality of the proteomics datasets but also to reliably assess KI rates from microscopy data (see points 12/13).

© 2024. Published by The Company of Biologists under the terms of the Creative Commons Attribution License (https://creativecommons.org/licenses/by/4.0/).

We think that questions regarding the general stability of fusion proteins with the different labeling enzymes is outside of the scope this manuscript and has been already discussed (May et al. 2020). We agree with the reviewer that this is an important point and we have addressed the topic of the stability of fusion proteins in the discussion.

Line 387:
"It needs to be noted that TurboID and especially miniTurboID protein fusions are reported to be less stable than BioID and could cause cell toxicity[68]. When probing expression of the fusion proteins on western blots, we indeed noticed that BioID2-fusions are higher expressed than miniTurboID, possibly due to higher stability of the fusion protein. We did not observe any signs of induced toxicity in our KI cells as cell growth and cell shape were comparable between KI cell lines and WT cells. However, this has to be tested for each protein tagged.
"

We generally did not observe morphological defects and differences in cell growth compared to WT when we cultivated the KI cells, suggesting that the cells we used for the experiments are rather healthy. In addition, many of the expected interactors for all APs were detected in the MS experiments, further suggesting the presented data is valid and transport pathways were intact when experiments were performed.

2. Sup Fig. 1, please add data for the 0h time point (missing here), there appears to be quite some non- Golgi staining for biotin for TurboID at 2h and 24h but it is hard to judge without a comparison for 0h biotinylation and WT cells.

We have added images of cells prior to addition of biotin (0 h time panel in Fig. S1C) as well as we have quantified the biotinylation staining for this time point in Fig. S1D). The background activity of TurboID was already discussed in the manuscript.
The non-Golgi staining is real signal as a major fraction of AP-1 localizes to ARF1 compartments in the periphery of the cell (we analyzed the role of AP-1 extensively in a different paper; see doi: 10.1101/2023.10.27.564143). However, upon fixation with PFA these peripheral compartments are disrupted and the AP-1 fraction at the Golgi is preserved better, thus AP-1 appears to be predominantly localized to the Golgi in fixed cells.

3. Fig. 4B: please include a blot for CLCa, this would also help appreciate why the amount of V5-TurboID seems to be enormously higher than that of the other enzymes (which is not commented by the authors).

To address this concern, we defrosted cells repeated the western blot and the quantification of KI efficiency and included the new data in the manuscript. It seems that BioID2 fusions are higher expressed for C-terminal and N-terminal fusions, although KI efficiencies are comparable to other fusion proteins. It could be that this is due to a higher stability of BioID2 compared to TurboID (May et al. 2020), we added a sentence to the discussion regarding this observation.

Line 387:
"It needs to be noted that TurboID and especially miniTurboID protein fusions are reported to be less stable than BioID and could cause cell toxicity[68]. When probing expression of the fusion proteins on western blots, we indeed noticed that BioID2-fusions are higher expressed than miniTurboID, possibly due to higher stability of the fusion protein. We did not observe any signs of induced toxicity in our KI cells as cell growth and cell shape were comparable between KI cell lines and WT cells."

We also tested two different CLCa antibodies, but both showed many unspecific bands making it difficult to interpret. We therefore want to omit the CLCa western blot from the manuscript, as the V5 blot by itself shows expression of the fusion proteins.

4. Fig. 3A: a negative control (WT cells treated with the same antibody/streptavidin, and

© 2024. Published by The Company of Biologists under the terms of the Creative Commons Attribution License (https://creativecommons.org/licenses/by/4.0/).

image processed with same parameters) is needed

The recommended controls are already shown for AP-1 in Figure 1D where we show WT cells treated with the same antibody and streptavidin. No signal for either V5 or streptavidin staining was observed. As the biotinylatation patterns are quite distinct for the different APs and no quantification was performed based on these images, we did not see any benefit in adding a negative control. Furthermore, the drastically different expression levels of the tested APs (AP-4 is 40-fold less abundant than AP-1 or AP-2), make it impossible to process all images with the same parameters and properly visualize the tested proteins.

5. lines 403/406: if the datasets obtained in this study are meant to serve as “starting point for studies aimed at unravelling the mechanisms of (…) AP complexes” then the MS data should be analyzed properly:

For the bioinformatic analysis of the MS data imputations were used with the Perseus package to replace missing values. As the number missing values in typical proximity-dependent biotinylation experiments is typically quite high and do not only represent missing completely at random and missing at random values (also because sample and negative control are quite different samples), imputation of missing values is typically not valid for such datasets as it strongly distorts the observed frequency distributions. If the authors believe this was a valid approach for their specific datasets, they should provide frequency distribution histograms of log2-transformed protein levels before and after imputations to prove their point. Otherwise I can recommend referring to Goeminne L et al., Anal. Chem. 2020, 92, 6278−6287 (see Fig. 1 for how imputation can strongly distort the dataset) and also Wolski W et al., J. Proteome Res. 2023, 22, 1092−1104 for alternative more suitable strategies to address missing values. The clearest hits will still be identified but the rest of the datasets will most probably be affected which will be important for future studies that may use the present datasets as a starting point.

We have exported the frequency distribution histograms (see below). We believe that for the majority of the data the imputation is not a big issue but for the AP-4 samples, a different analysis might be indeed beneficial. We therefore added a sentence in the method section and referred to the suggest publication to address this concern.

Line 758:
“For low abundance proteins (such as AP-4) sometimes many values need to be generated via imputation, which could lead to distortion of the data sets. In our case, we believe that it was not an issue, but other methods to replace missing values could be used[85].”

However, we believe that especially interactors and cargoes that were enriched against multiple APs are promising candidates for follow-up experiments. We have also stated in the discussion that the raw data is uploaded for re-analysis to ProteomeXchange.

Line 439:
“For re-evaluation all raw data were made available via ProteomeXchange with identifier PXD051393.”

© 2024. Published by The Company of Biologists under the terms of the Creative Commons Attribution License (https://creativecommons.org/licenses/by/4.0/).

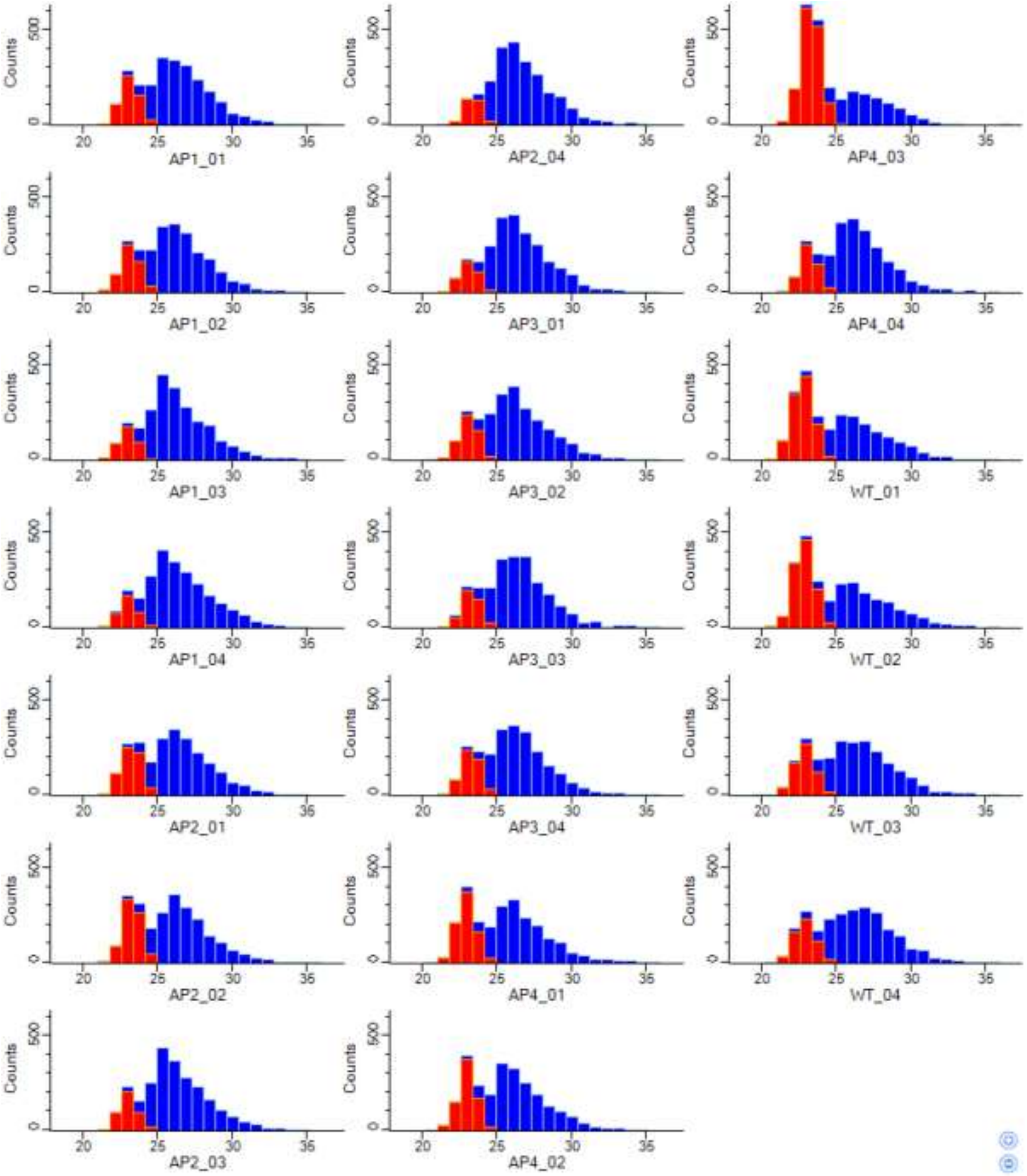

Figure for the reviewer: Frequency distribution histograms from the used samples. Imputated values are shown in red.

6. For all blots: please provide in the supplementary material images of the uncropped blots used to produce the figures and indicate the boxes that were used to quantify the signal for V5 and biotinylation.

We have provided the requested data along with our manuscript for submission. See Fig. S4.

7. For all blots: a loading control is missing and makes the quantifications unreliable, especially since the linearity of the quantified signals is not demonstrated.

The only quantification we performed from a western blot is in Fig. S1F. The quantification is based on the quantification that was performed to determine the efficiency of TurboID

© 2024. Published by The Company of Biologists under the terms of the Creative Commons Attribution License (https://creativecommons.org/licenses/by/4.0/).

and miniTurboID, when they first described the variants (doi: 10.1038/nbt.4201). As we work with mixed populations of cells, we believe that a V5 signal is a better quantification parameter to express labeling efficiency of the enzyme.

ADDITIONAL COMMENTS:
8. In the same line to point 7, since the quantification of the blots is based on ECL signals measured in triplicates each time from two separate blots (one for biotinylation, one for V5), the resulting data are not sufficient at all to make serious statistics (small amount of data points, technical methods prone to huge variations, ECL signals with limited linear range). Hence on Sup. Fig. 1F I recommend showing the bar for the average value and the corresponding three data points for each enzyme but no error bar and no mention of (non) significance. One can see by eye how the data look like and the statistics are of very limited relevance here.

We have changed the graph and removed the statistical analysis.

9. In the first paragraph of the result section, please clearly state what cell type was used in this study (in the present version it only appears in some of the figure legends and in the supplementary material).

We added the information about the cell type to the results part of the manuscript.

Line 101:
“To evaluate the performance of commonly used labeling enzymes, we used the CRISPR-Cas9 system to
genetically fuse APEX2, BioID2, miniTurboID and TurboID to the C-terminus of our chosen target AP1μA in HeLa cells.”

10. The description of the strategy to obtain a pool of knock-in cells is not detailed enough. Please indicate on your schemes (Fig. 1A and Fig. 4A) what you refer to as the “resistance cassette” is made of (Promoter / polyA signal or IRES / polyA signal of native locus?). For the sake of reproducibility I would also recommend providing in the supplemental material .gb files for the KI constructs as it is much easier than trying to reconstruct a similar plasmid following the instructions given in the supplementary material.

We have added the information that the resistance cassette is composed of a promoter, the resistance gene and termination sequence to the figure legend of Fig. 1. Moreover, we are depositing the TurboID plasmids on addgenes with sequences and writing a detailed protocol for tagging with fluorescent tags and TurboID. We would be happy to provide JCS with plasmids sequences if that is an acceptable format for a supplementary file.

11. Lines 101-109. The timeline of the experiments is not clear: G418 is applied 3 days after transfection until the cells are confluent and then the cells are analyzed. How many days is then G418 applied to the cells, how was it checked that this time window is long enough to kill all the cells that did not undergo HDR? If there is no data supporting this the estimated KI rates are not really telling.

The time frame is stated in the method section (G418 is applied for 7-10 days). As for all antibiotic selections we always test various concentrations of an antibiotic on WT cells of the selected cell type and select the lowest amount of antibiotic required to kill all non-edited cells (for HeLa cells 7 days are sufficient to kill all WT cell with the used concentration). For other cell types concentration and time frame can vary and testing various concentrations and times is always recommended.

12. For the quantification of the KI rates, the method description is too succinct to be reproduced by others (what parameters/threshold were used in the microscopy experiments). Rather than using microscopy I recommend using flow cytometry to measure KI rates, FC is much more powerful in term of unbiased quantification and will give the important information about the homogeneity of the expression of the fusion protein within

© 2024. Published by The Company of Biologists under the terms of the Creative Commons Attribution License (https://creativecommons.org/licenses/by/4.0/).

the selected the cell pool.

For quantification of the KI rate, the used approach is very straight forward, as cells are counted based on the presence of V5-signal which can be done directly at the confocal microscope and requires no image processing or similar. We unfortunately do not have easy access to flow cytometry. In addition, in our hands, cells expressing low abundant targets are hard to sparse from the WT population in flow cytometry experiments.

13. By reading the manuscript I understand that the KI rate is defined as the % of cells in which a V5 signal could be detected by microscopy after few days of G418 selection. The western blot data do not really correlate with the microscopy data (especially for the CLC KI cells), which may be due to different stability of the various fusion proteins. This point which is independent of the KI rate per se is only commented briefly in the discussion (line 352) but with a confusing wording: the KI rate (i.e. rate of foreign DNA integration in the host genome) can not be influenced by “lower stability of (…) fusion proteins”, these are two different things.

We agree with the reviewer, that the wording “KI rate” is confusing. To be more clear, we changed the wording to “KI efficiency” when we talk about the amount of cells that express the fusion protein.

14. Lines 110/111: strictly speaking since you have heterozygous cells the enzyme are not expressed at the endogenous levels of the target proteins but below endogenous levels.

We changed the text.

Line 117:
“Next, we wanted to compare the performance of the various labelling enzymes when expressed under the endogenous promoter.”

15. Line 132/134: “The low biotinylation rate of BioID2 makes it, in our opinion, unfavorable compared to the TurboID variants, as a high amount of biotinylation is crucial to enrich enough protein for the subsequent identification and quantification by mass spectrometry” the statement is rightfully presented as an opinion but can still be understood as a recommendation. I would recommend using a wording that makes it clear that this opinion is not a general recommendation as in other contexts the higher amount of biotinylation proved to lead to less specific datasets (see May D, Cells 2020 in which this was experimentally tested). In addition, the biotinylation signals shown here are before performing streptavidin pulldowns so the amount of biotinylation obtained with BioID2 would have been definitely enough for the subsequent MS analysis.

We added a sentence into the discussion, where we address this general concern about TurboID.

Line 371:
“Previous work has already demonstrated the TurboID variants outperform BioID or BioID2 regarding their labeling kinetics[9,27,28,68], however a larger effective labeling radius might lead to less specific data sets[68].”

16. Lines 195/196: Fig. 2E shows p-values of GO term enrichment, not fold changes, so the wording “more enriched” appears to be incorrect to describe the differences observed.

We changed the wording.

Line 201:
“While both data sets show enrichment of general GO terms such as *intracellular transport* or *vesicle- mediated transport*, AP-1-specific GO terms including *post-Golgi vesicle-mediated transport* or *endosomal transport*, are more frequent in the KI sample (Fig. 2E).”

© 2024. Published by The Company of Biologists under the terms of the Creative Commons Attribution License (https://creativecommons.org/licenses/by/4.0/).

17. In the comparison OE vs KI (Fig. 2G) quite a lot of hits are also enriched in the OE conditions. Do these proteins (possibly after applying a filtering with the cyt TurboID dataset) correspond to anything of significance for AP-1 biology (maybe cytosolic partners of the complex when it is not associated with the TGN)?

We have checked all significantly enriched proteins for OE and KI condition via Uniprot, and compiled lists of all proteins which matched the search criteria or could be of any significance to AP-1 biology. The results of this analysis are provided in the supplement tables 2 and 3 and depicted as Venn diagram in Fig. 2H. We excluded all cytoskeletal-associated proteins from the list, as we could not clearly say if they have any relevance for AP-1 biology, except those that are known to have regulatory functions in vesicular trafficking. We have stated in the method section, what parameters were chosen to search for potential interactors:
“These proteins were then screened with Uniprot to identify proteins that are either known to localize to either the Golgi and endosomal system (or to clathrin-coated pits in the case of AP-2), are thought to be involved in cellular trafficking, or are transmembrane proteins that are potential cargo proteins or could control trafficking events (e.g. kinases, phosphatases). All of those proteins that have at least one transmembrane domain were considered potential cargo proteins, all others were termed potential interactors. “.
We also provide the raw data for re-analysis.

18. Fig. 3A: any explanation why there is such a strong accumulation of biotinylated proteins in the nucleus of AP3A-muA-turboID expressing cells?

In general, we find AP-3 to be more cytosolic compared to other APs. This becomes apparent in live-cell experiments with Halo or SNAP-tagged AP-3. This could explain why there is more cytosolic/nuclear signal from biotinylated proteins. For all APs streptavidin-labeled biotinylated proteins were detected in the nucleus in microscopy experiments. A larger cytosolic fraction might correspond with a larger amount of biotinylated proteins in the nuclear area for AP-3.

19. Fig. 3E: the whole point of the manuscript is that one should not work with overexpressed proteins but for the validation presented here, the potential interactors are overexpressed. Since overexpression of cargo proteins has been described to potentially affect their transport route (see the example of SERINC3, a cargo of AP-4 in Davies A et al., 2018), is there any way to use antibodies against endogenous ITGB1, VAMP7 and SCYL2 instead of transient transfection of the GFP-tagged proteins?

We agree with the reviewer. However, the use of antibodies is not ideal for proteins working together with AP-1, as we have shown that the structures that harbour AP-1 are disrupted upon PFA fixation (see doi: 10.1083/jcb.202205107 and doi: 10.1101/2023.10.27.564143). KI approaches are out of the scope of this paper but we are indeed validating MS hits by generating KIs of some of the hits for a more biology focused manuscript.

20. Lines 211/213: “Taken together, our findings highlight that tagging the protein with the labelling enzyme endogenously increases the sensitivity of the proximity biotinylation and the MS measurement so that very transient but specific interactors, such as specific cargo proteins, can be identified.” The wording is misleading: it is actually the expression level (less than physiological vs overexpressed) that matters not how it is achieved (KI). As the authors very well describe in their introduction, overexpressing the target protein is not the recommended approach as this leads to artefacts such as mislocalization. Hence most PDB studies are not performed after transient transfection but use various strategies to achieve physiological levels. The KI approach is one possibility but not the only one.

We changed the sentence and focussed more on the expression level.

Line 219:

© 2024. Published by The Company of Biologists under the terms of the Creative Commons Attribution License (https://creativecommons.org/licenses/by/4.0/).

"Taken together, our findings highlight the importance of physiological expression levels of the TurboID- fusion protein. By tagging the AP1μA endogenously, we increases the sensitivity of the proximity biotinylation and the MS measurement so that very transient but specific interactors, such as specific cargo proteins, can be identified."

21. For the part on the AP-4 dataset interactors (lines 264-266), it would be good to compare the dataset obtained here with the dataset from Ref. 49, which used a completely different approach (the discussion would be a good place for this). In Supplementary Table 4, it appears that the comparison AP-4 vs. WT cells led to the identification of no significant hits for AP-4, this is not commented in the main text.

We added a couple of sentences to the discussion where we compare our findings to the ones in Ref.49.

Line 426:
"For AP-4 different approaches have been compared to identify AP-4 interactors[49]. Our results are comparable to data generated using an AP4ε-BioID fusion protein. Further, AP-4 cargoes could only be identified in a sensitive immunoprecipitation experiment using the AP-4 interactor Tepsin. This highlights general limitations of biotin-based proximity labeling approaches, as cargoes without or only short cytosolic tails may not have lysine residues as potential acceptors for biotinylation and cargoes which are luminal and are transported in vesicular or tubular transport intermediates are not accessible for biotinylation. For identification of such proteins, alternative approaches such as immunoprecipitation, cross-linking MS or immunoisolation of whole carriers/compartments would be required."

Regarding the AP-4 dataset, as one of the measurements was much worse than the other three (measurement 3; see frequency distribution provided earlier), the q-values are higher and thus t-test showed no significance. For the manuscript we relied on a different approach to characterize significance (log10 p-value>1.3, log2 fold change >1), which usually corresponds to a q-value of <0.05.

22. Line 368/369: "to prevent background biotinylation of TurboID, cells can be grown in biotin-free media 72h prior to the experiment". I would be very careful giving this advice without supporting data as a general fix for the background of TurboID as it may not be suited to all test systems (see e.g. Collins J. et al., JBC 1988 or Elahi A et al., J Immunol 2018). Labeling with heavy biotin and detection of heavy biotin- modified peptides is probably a safer approach to tackle this problem.

We want to thank the reviewer for this advice, we were not aware of this approach. We changed the manuscript accordingly.

Line 383:
"Practically, labeling with heavy biotin and detection of heavy biotin-modified peptides could be a solution to distinguish background biotinylation from induced biotin-labeling in TurboID experiments."

23. The limitations of the KI approach are not at all discussed: obviously 3'UTR are strongly modified (shortened) with the potential of losing important post-transcriptional regulation elements (which may be important for gene in e.g. cell differentiation test sytems). Any other potential limitation should be mentioned.

We included a sentence regarding this possible issue in the discussion.

Line 455:
"Nevertheless, expression levels of the edited protein should always be controlled when applying the presented strategy for C-terminal KIs, as the 3'UTR is shortened and might lose important gene-specific regulatory elements."

© 2024. Published by The Company of Biologists under the terms of the Creative Commons Attribution License (https://creativecommons.org/licenses/by/4.0/).

We always check expression levels of C-terminal KIs on a western blot. As for AP-1, we actually tested whether a strategy for C-terminal tagging with an exogenous polyA would lead to altered protein labels as compared to a LoxP-flanked cassette at the 3' end. No differences were observed.

24. The limitations of the PDB approach applied to the identification of cargo proteins for AP-mediated transport routes are also not addressed (e.g.: soluble cargo transported inside tubular vesicular structure are not accessible for biotinylation; transmembrane cargo proteins with short cytoplasmic tails may not have lysine residues as potential acceptors for biotinylation). A balanced discussion and mention of alternative/complementary approaches is needed.

We added a couple of sentences to the discussion. See answer to reviewer comment 21.

25. The MS data should be uploaded to a public repository

We uploaded the data to a public repository. The data is stored at ProteomeXchange and information is added to the manuscript.

Project Name: When less is more – A fast TurboID KI approach for high sensitivity endogenous interactome mapping
Project accession: PXD051393
Project DOI: Not applicable
Reviewer account details:
Username: reviewer_pxd051393@ebi.ac.uk
Password: FRez6W8n

*****
Reviewer 3 Advance Summary and Potential Significance to Field:

In their manuscript, Stockhammer et al. present a rapid approach for fusion of biotinylating enzymes at the N-and C-termini of proteins via knock-in at their endogenous genetic locus, enabling faster and more sensitive interactome mapping of any protein. Their method utilizes CRISPR-Cas9 homology-directed repair combined with an antibiotic selection cassette, eliminating the need for clonal propagation and analysis during cell engineering and selection. The authors demonstrate the effectiveness of their approach by tagging the C- termini of AP complexes 1-4, revealing both known interactors and cargo proteins, along with potential novel ones. They also introduce an endogenous editing strategy for N-terminal knock-ins, validated through mapping the interactome of the clathrin light chain. By comparing the interactomes of different AP complexes and clathrin, the authors identify specific interactors and biological cargoes associated with each protein complex.

Reviewer 3 Comments for the Author:
A protein's interactome is a key determinant of its biologic role. A common approach to mapping protein interactomes is proximity ligation, in which fusion to a labeling enzyme enables tagging of proximal, interacting proteins, which are then detected and quantified by mass spectrometry. Proximity ligation has historically faced challenges such as high assay noise from over-expression of labeling enzymes or complex genetic engineering requirements. A simplified method for generating endogenous genetic fusions with labeling enzymes would greatly aid in interactome mapping and functional understanding of proteins.

In their manuscript, Stockhammer et al. present a rapid approach for fusion of biotinylating enzymes at the N-and C-termini of proteins via knock-in at their endogenous genetic locus, enabling faster and more sensitive interactome mapping of any protein. Their method utilizes CRISPR-Cas9 homology-directed repair combined with an antibiotic selection cassette, eliminating the need for clonal propagation and analysis during cell engineering and selection. The authors demonstrate the effectiveness of their approach by tagging the

© 2024. Published by The Company of Biologists under the terms of the Creative Commons Attribution License (https://creativecommons.org/licenses/by/4.0/).

C- termini of AP complexes 1-4, revealing both known interactors and cargo proteins, along with potential novel ones. They also introduce an endogenous editing strategy for N-terminal knock-ins, validated through mapping the interactome of the clathrin light chain. By comparing the interactomes of different AP complexes and clathrin, the authors identify specific interactors and biological cargoes associated with each protein complex.

Comments and Concerns:

1. The presented approach may lack significant novelty. The supposed advancement of the approach is the inclusion of an antibiotic selection cassette, a technique commonly employed for selecting genetic knock- in cells.

We agree with the reviewer that the approach may not be fully novel. As we received enthusiastic feedback from the community when we initially presented our work, we decided to share it with everybody and we have so far shared our plasmids and expertise with many laboratories around the world. We believe that the paper has brought and explained the techniques closer to the community and has shown that the need for such a publication exist. As previously stated, we are processing submission of a methods paper to explain the process in detail and to deposit plasmids on Addgene. In addition to extensively explaining the advantages of endogenous tagging with TurboID, the provided AP interactor/cargo tables are a useful resource for the trafficking community.

2. Knock-in efficiency for each biotinylating enzyme is mentioned in the text but it is unclear exactly what these percentages represent. I.e., do state percentages indicate the percentage of alleles with integrations or the percentage of cells with at least one integration? Were these calculated pre- or post- selection with the antibiotic?

We always estimated KI rates after selection. Cells are stained with a V5 antibody and the percentage calculation is based on number of cells that show V5-staining/expression of the fusion protein. We included this information in the method section of the paper.

Line 623:
"*Quantification of KI efficiency*
To estimate the number of gene-edited cells after antibiotic selection, V5-immunostained cells for each KI cell line were screened for the presence of V5 signal using confocal light microscopy. 100 cells were screened for each cell line."

3. The authors do not provide validation that TurboID fusion to APμ subunits has no effect on AP complex formation and function. The authors should provide a supplementary figure demonstrating that AP complex function continues at normal levels.

We agree with the reviewer that testing the functionality of fusion proteins is important. We have provided such controls in a current preprint where we have endogenously tagged AP-1μA and AP-3μA with Halo and SNAP tags (see Stockhammer, Adarska et al., doi: 10.1101/2023.10.27.564143). Here we performed co-localization and functional studies using haploid HAP1 cells. In addition, a homozygous AP-1μA-Halo cell line does not display the phenotypic defects of an AP-1 KO (Also in Stockhammer, Adarska et al., doi: 10.1101/2023.10.27.564143). Furthermore, AP complex subunits are strongly enriched in the MS, suggesting that AP complexes assemble when the μ-subunit is TurboID-tagged. As all the AP complexes are structurally very similar, we believe tagging at the C-terminus of the μ subunit is a good strategy.

4. The STED data in figure 1 appears to demonstrate that there are many nanodomains for which biotinylation and expression for the enzyme do not overlap. Do the authors have an explanation for this observation?

We believe that membrane-associated biotinylated proteins will diffuse over time and some of the biotinylated proteins will not necessary always co-localize with AP-1. We noticed however, that when we overexpress the AP1μA-TurboID, we observed large areas where no co-localization is given are much more frequent than when we endogenously

© 2024. Published by The Company of Biologists under the terms of the Creative Commons Attribution License (https://creativecommons.org/licenses/by/4.0/).

expressed the fusion protein.

5. The authors chose 24 hour biotin labeling for interactome mapping. 24 hour labeling generates significantly more labeled proteins than 2 hour labeling based on western blot analysis (supplementary figure 1) and I am concerned that this increase in signal may reflect primarily higher levels of off-target labeling rather than on-target labeling. In a supplementary figure, the authors should include an analysis demonstrating that background/cytosolic biotin labeling is still minimal when TurboID labeling proceeds for 24 hours. Additionally, I wonder how MS results differ between 2 and 24 hours – are major known interactors missing?

We have prepared a 2 h biotinylation sample of the AP3µA-TurboID cells and could not find major differences between 2 h and 24 h labeling time. We emphasized in the discussion that shorter labeling times are possible when working with TurboID and relatively high abundant proteins.
In addition, we show in Fig. S2, that background labeling is only slightly increased with 24 h labeling time compared to 2 h of labeling.

6. The authors validate the possible novel interactors of AP-1 (ITGB1, VAMP7, and SCYL2) using co- localization by fluorescence microscopy. While there are points of co-localization between AP-1 and these proteins, there is no control condition, making it difficult to determine if co-localization events are biologically significant or simply chance overlap. The authors should perform a control with a non-interactor of AP1, and quantify and compare the colocalization of AP1 to each novel interactor and the control. Alternatively, the authors could perform a co-IP as validation.

We think additional experiments are outside of the scope of the here shown manuscript, especially since not all interactors and regulators will co-immoprecipitate with AP-1 as TurboID highlight the proximity and not necessarily direct interactions. Some interactions may also be weak or transient. This does not mean that the highlighted hits might not be relevant for AP-1 biology.
Nevertheless, we briefly want to explain why we think the shown proteins are valid AP-1 interactors/cargoes. We recently published a preprint in which we focus on AP-1 biology, AP-1 is on ARF1 compartments that mediate secretory transport and endocytic recycling (see Stockhammer, Adarska et al.;doi:10.1101/2023.10.27.564143), we believe that ITGB1 may be a cargo for endocytic recycling that uses these ARF1 compartments as it shows localization on tubular structures in a similar way to what we observe for transferrin. In our opinion, the overlap of SCYL2 with AP-1 is striking, and we believe no additional control is needed in this case to prove that it localizes to the same nano-domains AP-1 does.
For VAMP7, we stated in the manuscript why we think it might be relevant for AP-1 trafficking, although it has only been connected to AP-3 transport yet:
"VAMP7 is a component of a SNARE complex composed of syntaxin-8, syntaxin-7, VAMP7 and VTI1B that is involved in endosomal recycling of endocytosed material[60], and interestingly, we found all four members as potential AP-1 interactors."

7. In Figure 3, the authors identify interactors and/or cargo proteins of the AP1 complexes by comparing protein abundances between the AP complexes. For complexes that do not localize to the same subcellular space (e.g. AP1 and AP2), the landscape of non-specific labeling for these proteins may differ significantly, causing off-target proteins to become significantly enriched. Instead of comparing across AP complexes, the authors should have compared to a control that localizes to the same subcellular compartment and is likely to have a similar landscape of non-specific labeling.

There is never a perfect control and AP-specific interactors should be strongly enriched against any control. In fact, AP-1 and AP-3 localize to the same intracellular membranes (see Stockhammer, Adarska et al.;doi:10.1101/2023.10.27.564143), and the AP-1 vs AP-2 and AP-1 vs AP-3 MS shows highly similar results, indicating that AP-2 indeed can be used for identifying AP-1-specific interactors without localizing to the same compartments. Additionally, we believe that a control localizing to the exact same membrane would be

© 2024. Published by The Company of Biologists under the terms of the Creative Commons Attribution License (https://creativecommons.org/licenses/by/4.0/).

subtracting possible interesting hits.

8. Why do the authors validate the N-terminal fusion on clathrin as opposed to AP-1? I would be interested to see if similar results are achieved for N- and C-terminal fusions of AP-1.

Localization of the tag (C- and N-terminally) can impact protein function. AP μ-subunits are usually tagged C-terminally (see doi:10.1038/s41467-021-22267-0 or doi:10.1101/2023.12.06.570355) while CLCa is usually tagged N-terminally. We wanted to stick to what has been shown in the literature to not affect protein function.

9. The N-terminal TurboID fusion appears to generate higher background biotinylation (figure 4)? How do the authors explain this and are they concerned by this potentially higher background signal?

Clathrin is more abundant that AP-1 and is also involved in two different AP pathways (AP-1 and AP-2). We assume it has a larger cytosolic protein fraction compared to the APs, hence the stronger cytosolic background.

Second decision letter

MS ID#: JOCES/2024/261952

MS TITLE: When less is more - A fast TurboID KI approach for high sensitivity endogenous interactome mapping

AUTHORS: Alexander Stockhammer, Carissa Spalt, Antonia Klemt, Laila Benz, Shelly Harel, Vini Natalia, Lukas Wiench, Christian Freund, Benno Kuropka, and Francesca Bottanelli
ARTICLE TYPE: Tools and Resources

We have now reached a decision on the above manuscript.

To see the reviewers' reports and a copy of this decision letter, please go to: https://submit-jcs.biologists.org and click on the 'Manuscripts with Decisions' queue in the Author Area. (Corresponding author only has access to reviews.)

As you will see, the reviewers gave favourable reports but reviewers 2 and 3 have a few minor comments that will require amendments to your manuscript. I hope that you will be able to carry these out because I would like to be able to accept your paper.

Please ensure that you clearly highlight all changes made in the revised manuscript. Please avoid using 'Tracked changes' in Word files as these are lost in PDF conversion.

I should be grateful if you would also provide a point-by-point response detailing how you have dealt with the points raised by the reviewers in the 'Response to Reviewers' box. Please attend to all of the reviewers' comments. If you do not agree with any of their criticisms or suggestions please explain clearly why this is so.

Reviewer 1

Advance summary and potential significance to field

Overall, the paper presents a broadly important advance in proximity labeling by demonstrating the effectiveness of different enzyme and expression strategies. The proteomics datasets are exciting for researchers in the field of membrane traffic. I think the work is significant and the quality of the work is high. The revisions improve the manuscript readability.

© 2024. Published by The Company of Biologists under the terms of the Creative Commons Attribution License (https://creativecommons.org/licenses/by/4.0/).

Comments for the author

I have no additional Comments for the authors.

Reviewer 2

Advance summary and potential significance to field

In this revised version of the initial manuscript, the authors did a very good job in addressing my, and in my opinion the other reviewers', criticisms.

I however still have three small points that I would wish to have addressed before I can support publication without any reservation

Comments for the author

Most important:
My Previous point 7 was:
For all blots: a loading control is missing and makes the quantifications unreliable, especially since the linearity of the quantified signals is not demonstrated.

The response of the authors is:
The only quantification we performed from a western blot is in Fig. S1F. The quantification is based on the quantification that was performed to determine the efficiency of TurboID and miniTurboID, when they first described the variants (doi: 10.1038/nbt.4201). As we work with mixed populations of cells, we believe that a V5 signal is a better quantification parameter to express labeling efficiency of the enzyme.

I am still not convinced because:
The quantification is based on two separate blots: on blot 1 the streptavidin signal is estimated, on blot 2 the V5 signal is estimated. Fig. S1F shows the streptavidin signal from blot 1 normalized by the V5 signal from blot 2 and makes comparisons between enzymes (i.e. between different lanes on blot 1 normalized by the corresponding lanes from blot 2).

I respectfully disagree with the authors when they write: "a V5 signal is a better quantification parameter to express labeling efficiency of the enzyme" to dismiss the need of a loading control.

Reason for this is: while I agree that if the quantification was performed from a single blot (with two different colors in a fluorescence-based western blot) normalization with the V5 signal may suffice, a normalized value based on two signals coming from two separate blots absolutely needs a loading control to demonstrate that the lane-to-lane loading was consistent on blot 1 and on blot 2 (to exclude e.g. that the same amount of material was loaded across blot 1 but twice as much material was loaded in lane "mini-TurboID 2h" for V5 in blot 2). This may have been how it was performed in the original TurboID paper but this was at that time already wrong and should not have passed peer-review, and is certainly not a reason to become a standard.

Hence I would suggest either to add a loading control to these blots or alternatively if the samples are not available anymore to simply remove Fig. S1F and qualitatively describe these data.

Two minor points:

My previous point 5 about the bioinformatics analysis and the response of the authors concerning the validity of the imputation approach.

In their response the authors state:
We have exported the frequency distribution histograms (see below). We believe that for the majority of the data the imputation is not a big issue but for the AP-4 samples, a different analysis might be indeed beneficial. We therefore added a sentence in the method section and referred to the suggest publication to address this concern.

© 2024. Published by The Company of Biologists under the terms of the Creative Commons Attribution License (https://creativecommons.org/licenses/by/4.0/).

I see something else as to me in most samples, the imputed data in red do distort the normal distribution. But I agree that most identified hits are probably relevant.
Since the authors have the frequency distribution histograms after imputation it would be good to publish them in the supplementary material so that those who may like to use the datasets for further study can make an educated decision whether they first wish to re-analyze them or not.

My previous point 13 on the terms "KI rate"

I am fine with the term "KI efficiency" (that I would understand then as how much protein you get after KI) proposed by the authors but it was not updated in the sentence I originally pointed out: this is now on line 368 and reads "The lower KI rates of miniTurboID compared to TurboID might be result of lower stability(...)"

Reviewer 3

Advance summary and potential significance to field

Please see our original comments

Comments for the author

Thanks to the authors for providing thorough and thoughtful responses to each of my comments. With the additional clarifications, I am satisfied with the state of the manuscript.

However, I would like to emphasize that in the final version of the manuscript, the authors should make a concerted effort to ensure that their description of knock-in efficiency is as clear as possible. Many readers will expect “knock-in rates” to indicate the proportion of alleles with knock-ins rather than the proportion of cells with knock-ins (this is common in any fields that leverages genome-editing). The authors describe a knock-in approach that relies on antibiotic selection, which should result in 100% of cells that contain the knock-in after selection, yet they describe knock-in rates of 50-60%. This is highly confusing to anyone reading, particularly when the methods are not sufficiently detailed. Why do only 50-60% of selected cells exhibit protein expression? It seems that 100% of cells should have the knock-in at the DNA level, while only 50-60% successfully express the transgene. Knock-in rate into the DNA and successful expression of the transgene are completely separate metrics and the authors must make this clear.

Second revision

Author response to reviewers' comments

Reviewer 2

We understand the reviewer’s concern and we have removed panel F in Fig. S1.

The text now reads:

Line 141: Similar results were seen when levels of biotinylated proteins were probed on western blot (Fig. S1E).

Minor points

- A Fig. S5 showing the frequency distribution histograms after imputation is now provided.

- To make it clearer that the numbers express the % of cells where we can detect the fusion proteins we have simply stated that in the manuscript:

© 2024. Published by The Company of Biologists under the terms of the Creative Commons Attribution License (https://creativecommons.org/licenses/by/4.0/).

Line 628: Quantification of % of KI cells expressing the fusion proteins
To estimate the number of gene-edited cells after antibiotic selection that express the fusion protein, V5-immunostained cells for each KI cell line were screened for the presence of V5 signal using confocal light microscopy. 100 cells were screened for each cell line.

We changed the text accordingly and now use the term “cells that express the fusion protein” instead of “KI rate” or “KI efficiency”.

Reviewer 3
We thank the reviewer for the additional clarification. We now clearly state that we are simply estimating the % of KI cells expressing the fusion proteins.

We agree with the reviewer, that 100% of the cells should be edited on the DNA level, but it seems that only 50-60% expressing the transgene. We can also only speculate why that is the case. For example, we believe that we may be underestimating the % of edited cells as detection will depend on permeabilization and permeability of the cells to the antibodies. As for the N-terminal fusions, the expression of the fusion protein is also dependent on the efficiencies of transfection of the Cre recombinase. We are currently compiling a methods paper where we will have more space to discuss all these details.

Third decision letter

MS ID#: JOCES/2024/261952

MS TITLE: When less is more - A fast TurboID KI approach for high sensitivity endogenous interactome mapping

AUTHORS: Alexander Stockhammer, Carissa Spalt, Antonia Klemt, Laila Benz, Shelly Harel, Vini Natalia, Lukas Wiench, Christian Freund, Benno Kuropka, and Francesca Bottanelli
ARTICLE TYPE: Tools and Resources

Thank you for contacting the editorial office with your recent query. After investigation, I can confirm that the incorrect decision letter was sent to you. We sincerely apologise for any confusion this may have caused.

I am happy to tell you that your manuscript has been accepted for publication in Journal of Cell Science, pending standard publication integrity checks.

© 2024. Published by The Company of Biologists under the terms of the Creative Commons Attribution License (https://creativecommons.org/licenses/by/4.0/).
